# Impact of halogen chemistry on summertime air quality in coastal and continental Europe: application of the CMAQ model and implications for regulation

**DOI:** 10.5194/acp-19-15321-2019

**Published:** 2019-12-16

**Authors:** Qinyi Li, Rafael Borge, Golam Sarwar, David de la Paz, Brett Gantt, Jessica Domingo, Carlos A. Cuevas, Alfonso Saiz-Lopez

**Affiliations:** 1 Department of Atmospheric Chemistry and Climate, Institute of Physical Chemistry Rocasolano, CSIC, Madrid 28006, Spain; 2 Environmental Modelling Laboratory, Department of Chemical & Environmental Engineering, Universidad Politécnica de Madrid (UPM), Madrid, Spain; 3 National Exposure Research Laboratory, Environmental Protection Agency, Research Triangle Park, NC 27711, USA; 4 Office of Air Quality Planning and Standards, Environmental Protection Agency, Research Triangle Park, NC 27711, USA

## Abstract

Halogen (Cl, Br, and I) chemistry has been reported to influence the formation of secondary air pollutants. Previous studies mostly focused on the impact of chlorine species on air quality over large spatial scales. Very little attention has been paid to the effect of the combined halogen chemistry on air quality over Europe and its implications for control policy. In the present study, we apply a widely used regional model, the Community Multiscale Air Quality Modeling System (CMAQ), incorporated with the latest halogen sources and chemistry, to simulate the abundance of halogen species over Europe and to examine the role of halogens in the formation of secondary air pollution. The results suggest that the CMAQ model is able to reproduce the level of O_3_, NO_2_, and halogen species over Europe. Chlorine chemistry slightly increases the levels of OH, HO_2_, NO_3_, O_3_, and NO_2_ and substantially enhances the level of the Cl radical. Combined halogen chemistry induces complex effects on OH (ranging from –0.023 to 0.030 pptv) and HO_2_ (in the range of –3.7 to 0.73 pptv), significantly reduces the concentrations of NO_3_ (as much as 20 pptv) and O_3_ (as much as 10 ppbv), and decreases NO_2_ in highly polluted regions (as much as 1.7 ppbv); it increases NO_2_ (up to 0.20 ppbv) in other areas. The maximum effects of halogen chemistry occur over oceanic and coastal regions, but some noticeable impacts also occur over continental Europe. Halogen chemistry affects the number of days exceeding the European Union target threshold for the protection of human beings and vegetation from ambient O_3_. In light of the significant impact of halogen chemistry on air quality, we recommend that halogen chemistry be considered for inclusion in air quality policy assessments, particularly in coastal cities.

## 1 Introduction

Halogen (Cl, Br, and I) species and related processes have been known to deplete stratospheric ozone (O_3_) for several decades (Molina and Rowland, 1974; Farman et al., 1985). In the troposphere, the chemistry of halogens has been described in detail in recent reviews and references therein (Saiz-Lopez and von Glasow, 2012; Simpson et al., 2015), so it is just briefly outlined here. Halogen species affect the concentration of air pollutants by, e.g., directly destroying O_3_ (Reaction R1), indirectly decreasing O_3_ production by reducing NO_2_ (Reactions R2 and R3), and influencing the NO=NO_2_ ratio (Reactions R2 and R4) and the HO_2_=OH ratio (Reactions R5 and R6). The budgets of NO_*x*_ (NO CNO_2_) and HO_*x*_ (OH C HO_2_) also affect the formation of O_3_ (e.g., Sillman, 1999; Li et al., 2018).

(R1) $$\text{X}(\text{Cl},\;\text{Br},\;\text{I})+{\text{O}}_{3}\to \text{XO}+{\text{O}}_{2}$$

(R2) $$\text{XO}+{\text{NO}}_{2}\to {\text{XONO}}_{2}$$

(R3) $${\text{XONO}}_{2}+{\text{H}}_{2}\text{O}(l)\to \text{HOX}+{\text{HNO}}_{3}(l)$$

(R4) $$\text{XO}+\text{NO}\to \text{X}+{\text{NO}}_{2}$$

(R5) $$\text{XO}+{\text{HO}}_{2}\to \text{HOX}+{\text{O}}_{2}$$

(R6) HOX+hv→OH+X

The chlorine radical (Cl) initiates the oxidation of hydrocarbons (Reaction R7), including methane (CH_4_) and non-methane volatile organic compounds (NMVOCs), in a similar way to the OH radical, reducing the lifetime of CH_4_ and NMVOCs and leading to the formation of O_3_ in the presence of NO_*x*_ (Thornton et al., 2010).

(R7) $$\text{RH}+\text{Cl}\to \text{HCl}+{\text{RO}}_{2}$$

The combined effect of halogen chemistry on air quality is therefore complicated and depends heavily on local conditions, e.g., atmospheric compositions and oxidative capacity (Sherwen et al., 2017; Muñiz-Unamunzaga et al., 2018). Evaluation of the complex role of halogen chemistry in air quality requires the employment of advanced, high-resolution chemical transport models.

A number of modeling studies have been conducted to investigate the impact of individual halogen species on air quality. The chemistry of chlorine, mainly that of nitryl chloride (ClNO_2_), has been reported to increase the oxidation capacity and the formation of O_3_ in recent studies (Sarwar et al., 2012, 2014; Li et al., 2016; Sherwen et al., 2017; Sommariva et al., 2018). Bromine (Fernandez et al., 2014) and iodine (Saiz-Lopez et al., 2014) chemistry are reported to decrease the concentration of O_3_ over oceanic and terrestrial regions.

Only a few regional modeling studies have explored the combined influence of halogen chemistry on air quality. The first modeling study with combined halogen (Cl, Br, and I) chemistry was conducted by Sarwar et al. (2015), who used a hemispheric version of the Community Multiscale Air Quality (CMAQ) model (Byun and Ching, 1999; Byun and Schere, 2006; Mathur et al., 2017) and reported a decrease in surface O_3_ by ~ 15 % to ~ 48 % over the Northern Hemisphere by Br and I. Gantt et al. (2017) then utilized the CMAQ model to explore the role of halogen chemistry at a regional scale over the continental United States (US). While these studies focused on the Northern Hemisphere and the continental US, Muñiz-Unamunzaga et al. (2018) applied the full halogen chemistry version of CMAQ with a resolution of 4 km and reported an up to 5 ppbv decrease in O_3_ in the city of Los Angeles, California, US. Sherwen et al. (2017) used a global model, GEOS-Chem, in a regional configuration (with a grid size of 0:25° × 0:315°, ~ 25km× ~ 25 km) and predicted a large decrease in O_3_, on average 13.5 pptv (25 %) and as much as 28.9 pptv (45 %), in Europe. Sarwar et al. (2019) further updated the halogen chemistry in the CMAQ model and reported a reduction of 3 to 12 ppbv in annual average O_3_ over seawater, 3 to 6 ppbv over coastal areas, and 3 ppbv over inland areas by Br and I. These previous regional studies using various models (or versions of models) in different areas reported a large range of the halogen impact on O_3_, highlighting the uncertainty in this research field.

The regulation of air quality and the control of air pollutants emission in Europe started in the early 1970s and over 40 years of effort has successfully improved air quality throughout Europe (EEA, 2018a). Nonetheless, poor air quality persists in major cities like Madrid, Paris, and London (EEA, 2018a); this shows the need for continued air quality management and effective policy. Because the influence of halogens on air quality is uncertain and potentially has an impact on air quality management decisions, we have conducted regional simulations using the latest version of the CMAQ model implemented with comprehensive halogen sources and chemistry (Sarwar et al., 2019) to examine the overall effect of halogen species on air pollution over Europe. Considering that the grid size has a noticeable impact on air quality model predictions (Sommariva et al., 2018), we used a CMAQ model domain with 12 km horizontal resolution (higher than the previous studies on the halogen impact covering Europe) to simulate the levels of halogen species over Europe, examine the effect on the oxidation capacity and the concentration of air pollutants, and explore the potential implications for air quality policy related to NO_2_ and O_3_.

## 2 Method and materials

### 2.1 Data

The meteorological inputs for the CMAQ model were obtained from the Weather Research and Forecasting model (WRF 3.7.1) (Skamarock and Klemp, 2008; Borge et al., 2008a) as an offline input. The WRF model was initialized from global reanalyses from the National Centers for Environmental Prediction (NCEP) Global Forecast System (GFS) with a spatial resolution of 1°×1° and a temporal resolution of 6 h (available online at http://rda.ucar.edu/datasets/ds083.2/, last access: 22 November 2019), which was updated daily from NCEP global analyses with 0.5° resolution (available online at http://www.nco.ncep.noaa.gov/pmb/products/sst/, last access: 22 November 2019). NCEP’s ADP global upper-air (NCAR archive ds351.0) and global surface observations (NCAR archive ds461.0) were used to drive the simulation with a Newtonian relaxation technique in the WRF model.

Anthropogenic emissions for the year 2016 were taken from the 0.1°×0.1° gridded EMEP inventory (EMEP/CEIP, 2014). It should be noted that no anthropogenic chlorine sources are included in our emission inventory. The temporal profiles and vertical distribution needed to resolve the emissions were those used in the EuroDelta experiment (van Loon et al., 2007). Biogenic emissions were estimated using the Model of Emissions of Gases and Aerosols from Nature (MEGANv2.10) (Guenther et al., 2012). All emissions were gridded to our model domain, temporally allocated, and chemically speciated using the Sparse Matrix Operator Kernel Emissions (SMOKE) model version 3.6.5 (UNC, 2015; Borge et al., 2008b).

**Figure 1.**
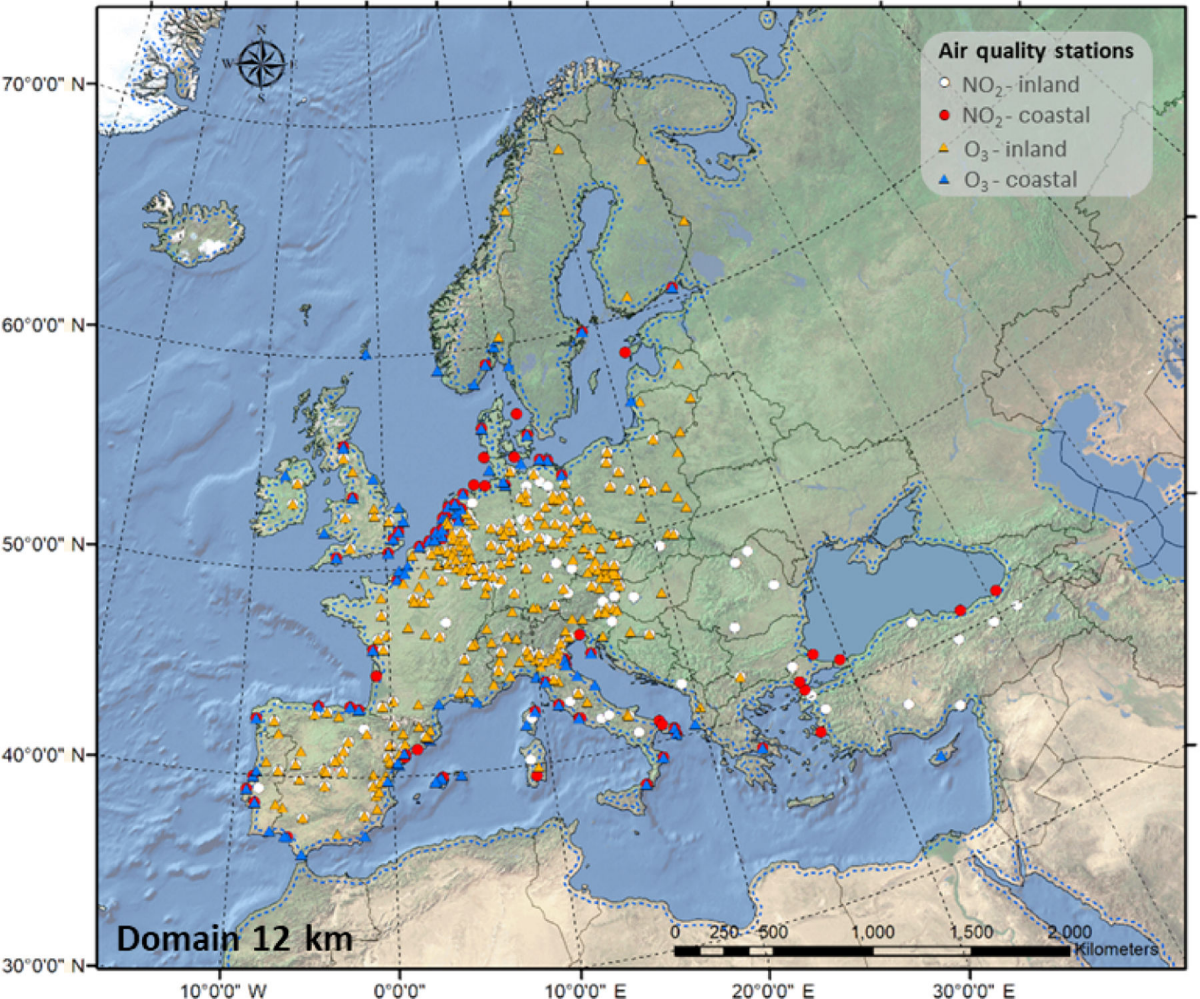
Geographic representation of the modeling domain and the air quality stations used for model evaluation.

In addition, we used measurement data on NO_2_ and O_3_ from over 400 background stations (traffic and industrial stations are not included) across Europe from the database Air-Base (public air quality database system of the EEA, 2018a) to compare the results of our simulation with observations (Fig. 1). Among these stations, 315 are located in inland areas (208 for NO_2_ and 266 for O_3_), and 125 are located in coastal areas (83 for NO_2_ and 93 for O_3_).

### 2.2 Modeling system

The CMAQ model is widely used and includes comprehensive representations of many essential atmospheric processes. The skill of the model in reproducing observed air quality has been demonstrated in many previous studies (Foley et al., 2010; Appel et al., 2013, 2017; Mathur et al., 2017), including applications over Europe (Borge et al., 2008a; Appel et al., 2012; Solazzo et al., 2017). CMAQ version 5.2 (https://www.epa.gov/cmaq, last access: 22 November 2019; https://doi.org/10.5281/zenodo.1167892, US EPA ORD, 2017) containing the Carbon Bond chemical mechanism (Appel et al., 2017) with halogen chemistry was used in this study. We updated the chlorine chemistry of Sarwar et al. (2012) and implemented it in CMAQ version 5.2 (Table S1 in the Supplement). The heterogeneous hydrolysis of dinitrogen pentoxide (N_2_O_5_) can produce ClNO_2_ and nitric acid (HNO_3_) in the presence of particulate chloride. In the absence of particulate chloride, the heterogeneous hydrolysis of N_2_O_5_ produces only HNO_3_. Sarwar et al. (2015) implemented the initial bromine and iodine chemistry in CMAQ, which has recently been updated by Sarwar et al. (2019). The updates include revising the gas-phase bromine and iodine reactions, incorporating several heterogeneous reactions of bromine and iodine species, incorporating several aqueous-phase bromine reactions, and revising the iodine and bromine emissions. We combine the updated chlorine, bromine, and iodine chemistry with the Carbon Bond chemical mechanism and use it in this study.

### 2.3 Simulation setup

A detailed description of physics and other model options (Table S2) can be found in de la Paz et al. (2016). The CMAQ model is applied over a domain covering the entirety of Europe (Fig. 1) with 12 km horizontal resolution. The vertical extent of the model extended from the surface to 100 mbar and contained 35 layers with an average surface layer thickness of approximately 20 m. The CMAQ chemical transport model is configured to use the piecewise parabolic method to describe advection processes, the Asymmetric Convective Model (version 2) to describe vertical diffusion processes, and the multiscale method to describe horizontal diffusion processes. Gas-phase chemistry, aqueous chemistry, aerosol processes, and dry and wet deposition were also included. The Rosenbrock solver was used for gas-phase chemistry.

The study was completed for the month of July 2016 with a spin-up period of 30 d. We performed three simulations to isolate the effect of halogen chemistry on air quality (in brackets is the name of the scenario used hereafter):

1. base model without halogen chemistry (BASE),
2. BASE with chlorine chemistry (CL), and
3. CL with Br and I chemistry (HAL).

The BASE model simulation includes the Carbon Bond 2015 chemical mechanism but does not contain any halogen chemistry, and only HNO_3_ is produced from the heterogeneous hydrolysis of N_2_O_5_. The CL simulation contains the Carbon Bond chemical mechanism with chlorine chemistry and considers ClNO_2_ and HNO_3_ production from the heterogeneous uptake of N_2_O_5_ on the aerosol surface. The HAL simulation contains the Carbon Bond chemical mechanism with full halogen chemistry and produces ClNO_2_ and HNO_3_ from the heterogeneous uptake of N_2_O_5_ on the aerosol surface.

Boundary conditions for the model were derived from the hemispheric CMAQ simulations (Mathur et al., 2017). Three different annual simulations were conducted using the hemispheric CMAQ model for 2016: the first simulation used the Carbon Bond chemical mechanism without any halogen chemistry, the second simulation used the Carbon Bond chemical mechanism and the chlorine chemistry, and the third simulation used the Carbon Bond chemical mechanism and the full halogen chemistry. Results from the corresponding hemispheric CMAQ simulation were used to generate boundary conditions for the BASE, CL, and HAL simulations. Therefore, the difference between CL and BASE simulations represents the impact of the chlorine chemistry on air quality, and the difference between HAL and BASE simulations represents the effect of halogen chemistry on air quality.

## 3 Results and discussion

### 3.1 Evaluation of model performance

The performance of the CMAQ model in simulating air quality over Europe is evaluated using observation data collected from > 400 measurement stations. We separate the stations into coastal (within 24 km of the coast) and continental stations (Fig. 1). Table 1 presents the statistics of the model performance for O_3_ and NO_2_ for BASE and HAL simulations.

The BASE and HAL simulations generally reproduce the concentration levels and the temporal variations of O_3_ and NO_2_ both at coastal and continental stations. The correlation coefficients between simulations and observations (Fig. S1 in the Supplement) show that CMAQ satisfactorily reproduces the variation of O_3_ and NO_2_ over most of Europe, especially the coastal regions (> 0:6 for O_3_ and > 0:4 for NO_2_). The BASE simulation underpredicts O_3_ compared to observations at both coastal and continental stations (Table 1), possibly due to the uncertainty of the NMVOC emission inventory (Sherwen et al., 2017) and the underestimated NO_*x*_ (Table 1). The HAL simulation slightly improves the correlation coefficient of O_3_ but decreases the average level of O_3_ compared to the BASE case. Diurnal variation plots (Fig. S2) suggest that both BASE and HAL simulations can reproduce the temporal patterns of O_3_.

The BASE simulation underpredicts NO_2_ compared to observations at both coastal and continental stations (Table 1). Such an underestimation of NO_2_ can occur for many reasons, including (1) positive artifacts of NO_2_ monitors (Jung et al., 2017), (2) underestimation of NO_*x*_ in the emission inventory, and (3) rapid transformation of NO_2_ into HNO_3_ in the model compared to the real atmosphere. Model performance is reasonable as the NO_2_ underestimation is relatively small. The HAL case predicted very similar NO_2_ concentrations (Table 1).

Overall, the evaluation of CMAQ results over Europe demonstrates that the model is capable of reproducing the levels of atmospheric chemical species and can be used to investigate the impact of halogen chemistry on air quality over Europe. It also suggests that the incorporation of halogen chemistry changes the model performance for O_3_ concentrations by a small margin without a noticeable impact on the model performance for NO_2_.

### 3.2 Simulated halogen species

Average surface concentrations of the inorganic halogen species predicted in the HAL simulation over the ocean are summarized in Table 2. HCl is the dominant chlorine species, with an average level of 247.9 pptv representing over 96% of the total inorganic chlorine (Cl_*y*_ ), while the average ClNO_2_ and HOCl is 4.9 pptv (1.9 %) and 3.8 pptv (1.5 %), respectively, with the remaining species contributing less than 1 %. The Br_*y*_ species are relatively evenly partitioned, with HOBr (1.06 pptv, 27.0 %), BrCl (0.71 pptv, 18.2 %), BrNO_3_ (0.67 pptv, 16.9 %), HBr (0.66 pptv, 16.8 %), and Br_2_ (0.33 pptv, 8.4 %) being the abundant species, while the remaining species contribute < 5 %. HOI (5.1 pptv, 52.0 %), INO_3_ (2.8 pptv, 28.7 %), and IO (1.0 pptv, 10.3 %) contribute over 90% of I_*y*_ over the ocean, while the remaining species contribute ~10 %. The predicted average concentrations of the critical halogen radicals, Cl, BrO, and IO, are 2.0×10^−4^, 0.03, and 0.4 pptv, respectively, over the ocean in Europe.

**Figure 2.**
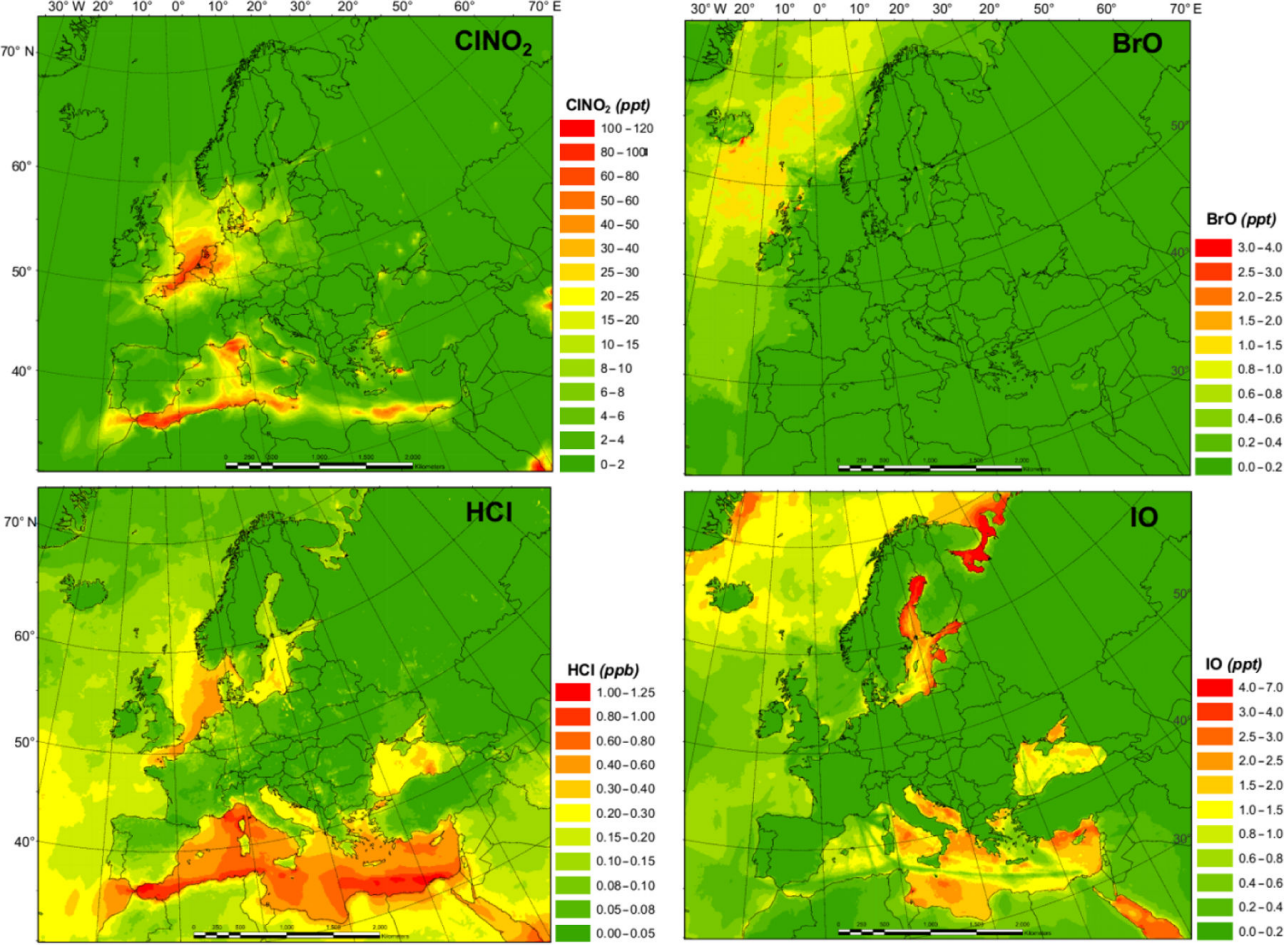
Monthly average ClNO_2_, HCl, BrO, and IO concentration in the HAL simulation.

The spatial distributions of key halogen species are shown in Fig. 2. The HAL simulation with full halogen chemistry simulates generally higher ClNO_2_ levels (with the highest monthly average value of 113.0 pptv) along the coast of the Mediterranean Sea and the North Sea with some influence into continental Europe, especially in Germany. The simulated HCl shows a similar pattern to that of ClNO_2_ but with a much higher concentration (> 10 times higher). The predicted BrO levels over Europe are low (average value ~0.17 pptv), with the largest predicted value occurring within the Arctic Circle, while GEOS-Chem predicted > 1.0 pptv of BrO in the Mediterranean Sea (Sherwen et al., 2017). The predicted IO peaks over the Mediterranean region with a maximum value of 6.9 pptv.

Direct measurements of halogen species are very scarce and not available for the period covered in the present study (July 2016). Since a direct comparison is not possible, here we present a comparison of the simulated concentrations with observations from previous studies (Table 3) to provide an approximate assessment of the representation of halogen species in the HAL simulation of the CMAQ model over Europe.

Numerous ClNO_2_ measurements have been reported around the globe, which show that ClNO_2_ is ubiquitous in the boundary layer, with maximum values ranging from hundreds to thousands of parts per trillion by volume in polluted coastal (Osthoff et al., 2008; Wang et al., 2016) and continental regions (Tham et al., 2016; Thornton et al., 2010). A few campaigns have been conducted in Europe. Phillips et al. (2012) reported a maximum value of 800 pptv for ClNO_2_ in Hessen, Germany, where CMAQ predicts a concentration of 273 pptv. Bannan et al. (2015) observed a peak value of 724 pptv in London where CMAQ predicts a concentration of 802 pptv. Simulations with the GEOS-Chem model (Sherwen et al., 2017) reported maximum values of 110 and 140 pptv in Hessen and London, respectively. Several field campaigns have been conducted in Weybourne in the past few years to measure ClNO_2_. Sherwen et al. (2017) reported a peak concentration of 946 pptv. Bannan et al. (2017) reported a peak value of 65 pptv, and Sommariva et al. (2018) reported a peak value of 1100 pptv in summer, 75.6 pptv in autumn, and 733 pptv in winter. CMAQ simulated a maximum of 373 pptv at that location, while GEOS-Chem predicted 458 pptv. Sommariva et al. (2018) also reported measurements of ClNO_2_ in Leicester with a maximum value of 274 pptv in spring, 74.2 pptv in summer, and 248 pptv in winter, with a peak value at Penlee Point of 922 pptv. CMAQ predicted a maximum of 274 pptv in Leicester and 319 pptv at Penlee Point. Eger et al. (2019) conducted shipborne observations of ClNO_2_ in the Mediterranean Sea and reported up to 600 pptv ClNO_2_ during their campaign, which is similar to the prediction of the present study.

The observed levels of HCl in Europe range from < 100 to 5000 pptv (Hossaini et al., 2016, and the references therein). The CMAQ model predicted monthly average concentrations of HCl of 6.3 to 1249 pptv, which is similar to the observation ranges. GEOS-Chem (Sherwen et al., 2017) predicted a maximum of 12 pptv for HCl, which is significantly lower than the measurements in Europe.

BrO measurements have been reported at ground-based sites and during the ship cruises, which generally demonstrate a range of 0.5 to 2.0 pptv maximum values for land measurements and 3.0 to 3.6 pptv for ship measurements (Saiz-Lopez and von Glasow, 2012). BrO observations have been reported at several coastal sites in Europe. BrO levels of up to 6.5 pptv (Saiz-Lopez et al., 2004) and 7.5 pptv (Mahajan et al., 2009) were reported in Mace Head and Brittany, respectively. CMAQ predicts 10.1 and 0.4 pptv at those locations, which are lower than the measurements. Sherwen et al. (2017) also predicted small values, with a maximum of 0.8 pptv in Mace Head and 0.5 pptv in Brittany. An extremely high level of BrO, ~ 100 pptv, was observed over the Dead Sea (Matveev et al., 2001; Holla et al., 2015). CMAQ is not able to reproduce such a high level of BrO due to the lower bromide content in typical ocean water (which was used in the present study for the Dead Sea) compared to the exceptionally high bromide content in the Dead Sea (Tas et al., 2006; Sarwar et al., 2015).

Global measurements show that the IO levels observed by ground-based campaigns were generally between 0.2 and 2.4 pptv, while those by ship measurement were ~ 3.5 pptv (Saiz-Lopez and von Glasow, 2012). Observations of IO have also been conducted in Europe. Maximum IO levels of 4.0–50.0 pptv were measured at Mace Head (Allan et al., 2000; Commane et al., 2011). CMAQ predicts a value of 3.9 pptv at Mace Head, while GEOS-Chem predicted a value of 0.6 pptv (Sherwen et al., 2017). In Brittany, up to 7.7–30.0 pptv of IO were observed by Bitter et al. (2005) and Furneaux et al. (2010). CMAQ predicts 1.1 pptv of IO at Brittany and Sherwen et al. (2017) predicted 0.07 pptv. A maximum IO concentration of 2.0 pptv was reported in Dagebüll (Peters et al., 2005), and CMAQ predicts 9.0 pptv at that site, while GEOS-Chem predicted 1.8 pptv (Sherwen et al., 2017). Prados-Roman et al. (2015) reported the level of IO during a ship-based campaign in the range of < 0:4 to > 1:4 pptv (daytime average) around the globe and 0.4 to 0.5 pptv (daytime average) in the south of Spain and the west of Africa (over the Atlantic); the present study predicted 0.4 to 2.0 pptv (daytime average) of IO over those areas.

### 3.3 Influence of halogen chemistry on the atmospheric oxidation capacity

**Figure 3.**
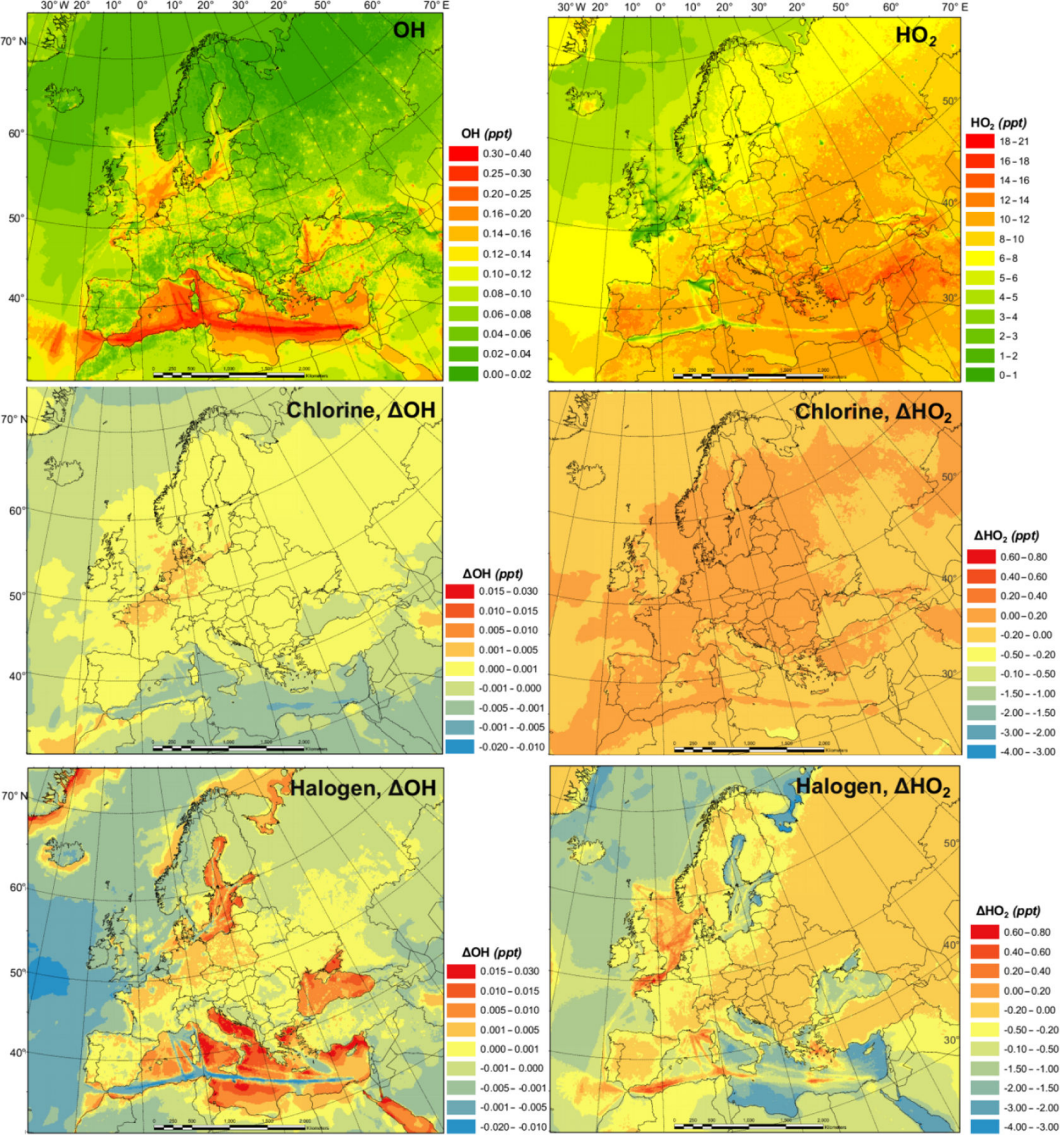
Monthly average OH and HO_2_ concentration in the BASE simulation and changes due to chlorine (CL) and full halogen chemistry (HAL).

Figure 3 shows the monthly average concentrations of the OH and HO_2_ radicals predicted by the BASE simulation as well as the impact of chlorine chemistry (CL-BASE) and the full halogen chemistry (HAL-BASE) on the simulated OH and HO_2_ levels. In the BASE simulation, the highest OH concentration levels are predicted over the oceans, especially along ship tracks, with a maximum value of 0.38 pptv (~ 9.5×10^6^ molecule cm^−3^). The chlorine chemistry slightly increased the OH level over most of the domain by up to 0.0041 pptv (1.0×10^5^ molecule cm^−3^). The impact of the halogen chemistry has competing effects on OH concentrations, with a maximum increase of 0.030 pptv (7.5×10^5^ molecule cm^−3^) and a reduction of as much as 0.023 pptv (5.8×10^5^ molecule cm^−3^). The BASE simulation predicts the highest values of HO_2_ over the Mediterranean Sea, with a maximum value of 21 pptv. The chlorine chemistry increases the HO_2_ level in the areas of elevated ClNO_2_ predictions (Figs. 2 and 3). The further addition of halogen chemistry lowers the HO_2_ as much as 3.7 pptv and increases HO_2_ up to 0.73 pptv compared to the BASE simulation. The overall difference of HO_2_ because of halogens was –0:59 pptv in the European domain. The effect of halogen chemistry on OH and HO_2_ is the combined effect of the following four pathways: (1) conversion of HO_2_ to OH via XO (Reactions R5 and R6), in which HO_2_ decreases and OH increases; (2) reduction of O_3_ (Reaction R1 and Fig. 7) and the reduced production of OH by O_3_ photolysis, in which both OH and HO_2_ decrease; (3) increase in NO_2_ (Reaction R4 and Fig. 7) and the enhanced consumption of OH by the reaction with NO_2_, in which both OH and HO_2_ decrease; and (4) increased oxidation of VOCs due to halogens in which both OH and HO_2_ increase. Pathway (1) leads to the increase in OH and decrease in HO_2_ over coastal areas, the Mediterranean Sea, and the Baltic Sea (except the ship tracks). Pathway (2) results in a decrease in OH and HO_2_ over the remote ocean. Pathways (2), (3), and (4) result in a decrease in OH and an increase or decrease in HO_2_ along the ship tracks in the Mediterranean Sea.

Sarwar et al. (2015) reported a small overall decrease in OH (1 %) and a significant decrease in HO_2_ (11 %) in the Northern Hemisphere due to bromine and iodine chemistry. Their results suggest a considerable reduction of the HO_2_=OH ratio, which is consistent with the present study. Muñiz-Unamunzaga et al. (2018) found a slight increase in diurnal OH (1 %–2 %) and a noticeable decrease in HO_2_ (4 %), leading to a decrease in HO_2_=OH in Los Angeles, California. Sherwen et al. (2017) suggested that OH was reduced across their European domain due to halogen chemistry and concluded that the shift of HO_2_ to OH by XO could not compensate for the decrease in OH due to the loss of O_3_. Another GEOS-Chem study, however, predicted an increase in OH over the Mediterranean Sea (Stone et al., 2018). The discrepancy among previous studies and between those works and the present one is difficult to deduce and requires further investigation. Several possible causes could lead to different simulated levels of halogens and their impact on oxidants, including the different mechanisms of producing and recycling halogen species (Sarwar et al., 2019), spatial resolution (Sommariva et al., 2018), emission inventory (Wang et al., 2019), and different spatiotemporal scale of interest (Stone et al., 2018).

**Figure 4.**
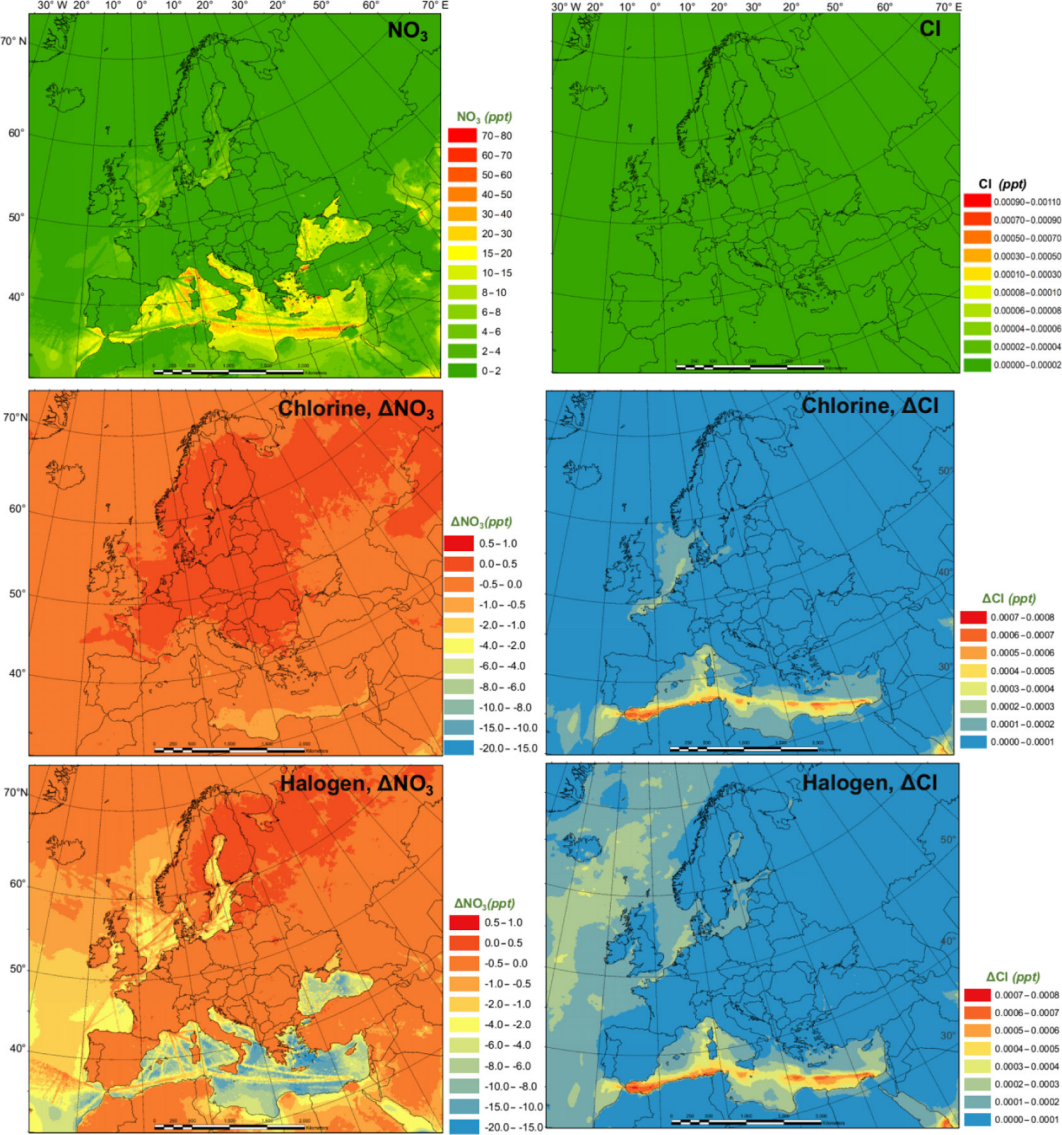
Monthly average NO_3_ and Cl radical concentrations in the BASE simulation and changes induced by chlorine (CL) and full halogen chemistry (HAL).

Figure 4 presents the monthly average prediction of NO_3_ and Cl radicals in the BASE scenario and the influence of chlorine (CL-BASE) and halogen chemistry (HAL-BASE) on the levels of NO_3_ and Cl. The BASE simulation predicted relatively high NO_3_ concentrations over the Mediterranean Sea along the busy shipping tracks. Although concentrations as high as 71 pptv are found, the majority of oceanic regions have concentrations in the range of 10 to 40 pptv and between 0 and 4 pptv over land. The chlorine chemistry slightly increases the NO_3_ radical due to the increase in O_3_ (see Sect. 3.4). In contrast, the halogen chemistry considerably reduces NO_3_ concentrations by as much as 20 pptv in the nighttime over the Mediterranean Sea. Muñiz-Unamunzaga et al. (2018) reported a 20 %–50% (2 to 4 pptv) decrease in the NO_3_ radical in Los Angeles, California, when considering the halogen chemistry.

In the BASE simulation, the Cl concentration was negligible because there was no relevant chlorine source incorporated in the CMAQ model. The CL simulation contains the production of ClNO_2_ and its subsequent photolysis, which increases the Cl concentration to as high as 7.0×10^−4^ pptv (~ 1.75×10^4^ atom cm^−3^). The HAL simulation predicted a very similar magnitude and spatial distribution of chlorine concentration. Sherwen et al. (2017) reported Cl concentrations less than 1.4×10^4^ atom cm^−3^ (~ 5.6×10^−4^ pptv) over Europe, comparable to our prediction. Hossaini et al. (2016) reported more than 1.0×10^4^ atom cm^−3^ ( 4.0 10^−4^ pptv) of chlorine over Asia, Europe, and North America, with a maximum of 8.5×10^4^ atom cm^−3^ (~ 3.4×10^−3^ pptv), using a global chemical transport model (TOMCAT) that incorporated chlorine sources from sea salt dechlorination, coal and biomass burning, oxidation of natural and anthropogenic chlorocarbon, and heterogeneous reactions on sea salt and sulfate aerosol. In their study, Hossaini et al. (2016) used the Reactive Emission Inventory of Chlorine (Keene et al., 1999), which Wang et al. (2019) reported to be unrealistic for present-day applications.

The current study and previous studies simulated a broad range of surface Cl concentrations, although they were all within the scope of the reported observed (observation-based calculation) values of 10^3^ to 10^5^ atom cm^−3^ (~ 4.0×10^−5^ to 4.0×10^−3^ pptv) according to the review of Saiz-Lopez and von Glasow (2012). In light of the considerable variation of observed and model-predicted Cl levels, further study may be needed to comprehensively evaluate the significant role of Cl in the troposphere.

**Figure 5.**
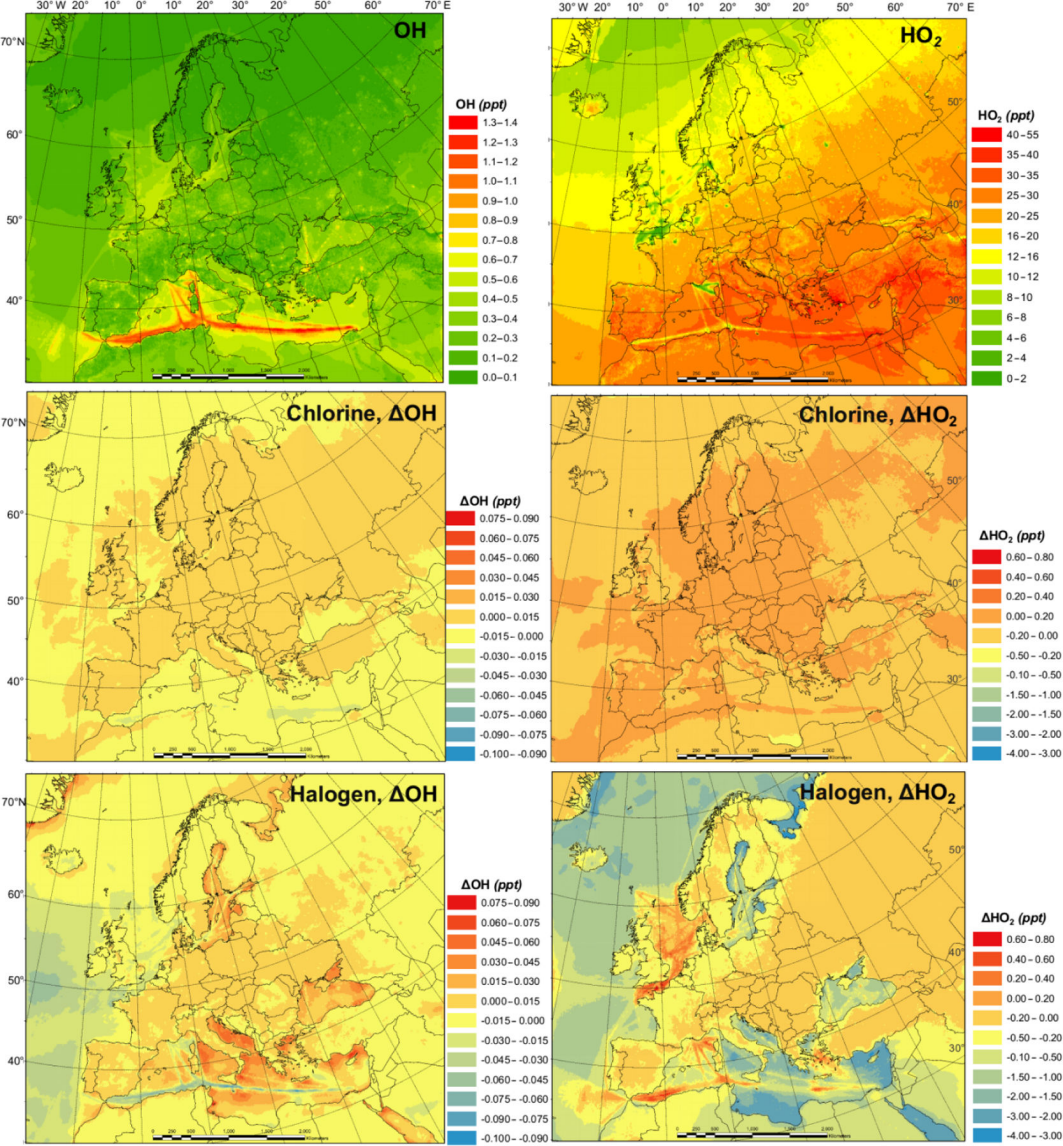
Monthly average of daily maximum concentrations of OH and HO_2_ in the BASE simulation and changes due to chlorine (CL) and full halogen chemistry (HAL).

**Figure 6.**
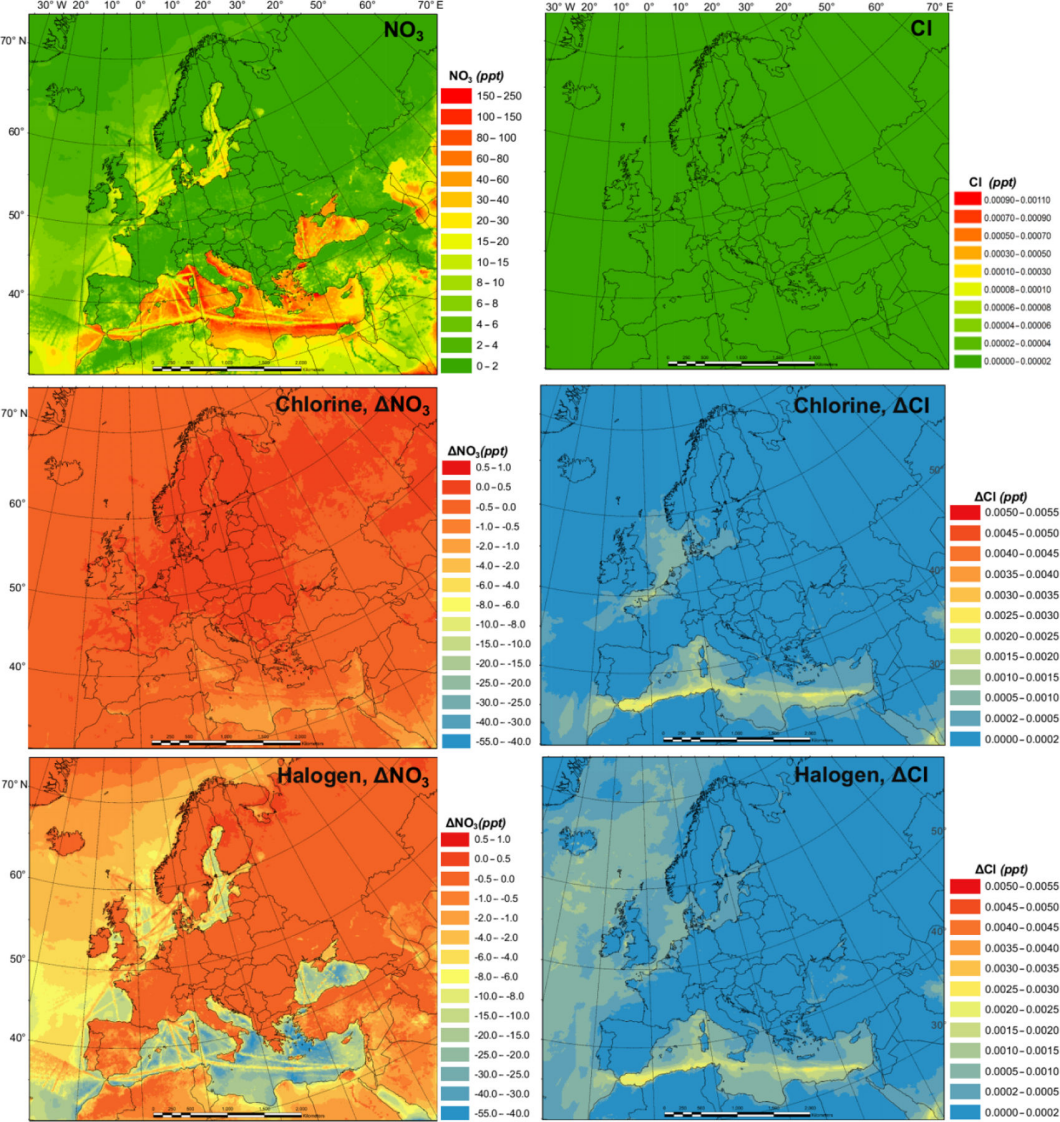
Monthly average of daily maximum concentrations of NO_3_ and the Cl radical in the BASE simulation and changes induced by chlorine (CL) and full halogen chemistry (HAL).

Figures 5 and 6 demonstrate the monthly average of the daily maximum concentrations of OH, HO_2_, NO_3_, and Cl in the BASE simulation and also the impact of chlorine (CL-BASE) and the halogen chemistry (HAL-BASE). The maximum values of OH, HO_2_, and Cl were predicted during the daytime, but they peak at different hours with Cl in the early morning and OH and HO_2_ later in the day, while the highest levels of the NO_3_ radical were simulated during nighttime.

The monthly averages of the daily maxima of OH, HO_2_, NO_3_, and Cl (Figs. 5 and 6) have similar spatial patterns and higher concentrations (or changes in concentration) compared to those of the monthly averages (Figs. 3 and 4). The monthly average of the daily maximum OH (HO_2_ and NO_3_) radical is 3.7 (2.5 and 3.4) times the monthly average OH (HO_2_ and NO_3_) concentration in the BASE simulations, while both the monthly average of the daily maximum and monthly average Cl show negligible values. The monthly average of daily maximum changes in the OH (HO_2_) concentration due to the chlorine and halogen chemistry has a magnitude of −0.10 to 0.09 pptv (−4.0 to 0.8 pptv), which is wider than (the same as) that of the monthly averages, i.e., −0.020 to 0.03 pptv (−4.0 to 0.8 pptv). For the NO_3_ (Cl) radicals, the magnitude of changes in the monthly average is −20 to 1.0 pptv (0.0 to 0.0008 pptv), while that in the monthly average of daily maximums is −55 to 1.0 pptv (0.0 to 0.0055 pptv).

We also examine the diurnal variations of the four radicals in the BASE and HAL scenarios (Figs. S3 and S4). Halogens have a small effect on the diurnal pattern of OH. HO_2_ is reduced by halogens, especially in the midday. The NO_3_ radical is strongly decreased throughout the night after the addition of halogens. The Cl atom is released by the halogen chemistry in the early morning. The significant effects of halogen chemistry on the diurnal variation of OH, HO_2_, NO_3_, and Cl radicals highlight the role of halogen chemistry in regulating the atmospheric oxidation capacity throughout the day, with the highest effect on Cl in the early morning, maximum effects on OH and HO_2_ in daytime, and the largest effect on NO_3_ at night.

### 3.4 Impact of halogen chemistry on regulated gaseous air pollutants

**Figure 7.**
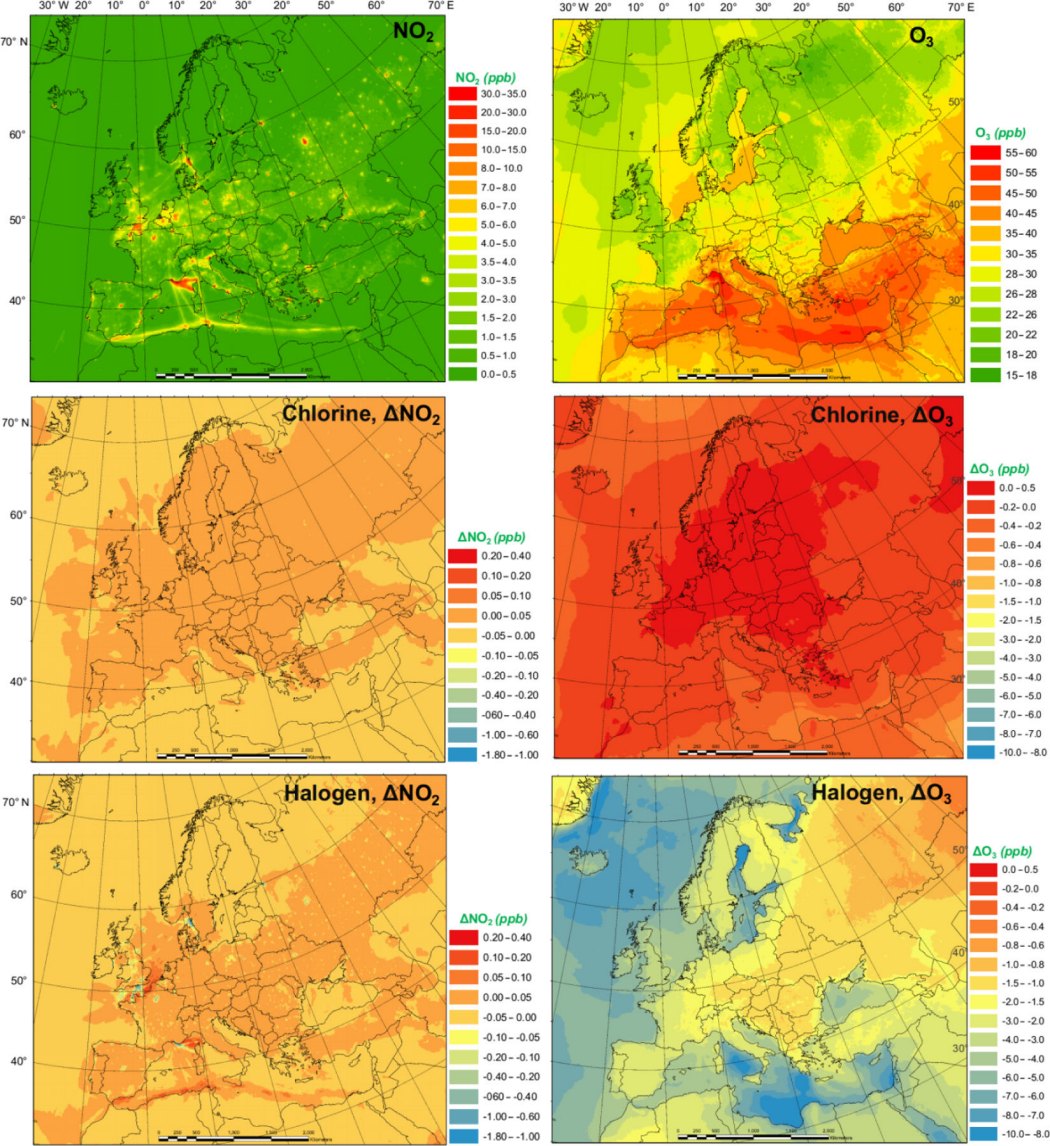
Monthly average NO_2_ and O_3_ concentration in the BASE simulation and changes induced by chlorine (CL) and full halogen chemistry (HAL).

The monthly average modeled NO_2_ and O_3_, two major gaseous air pollutants in Europe, and the effect of chlorine and halogen chemistry on the two regulated gaseous species are shown in Fig. 7. The BASE simulation produced many hot spots of NO_2_ over Europe in the vicinity of major cities and ship trajectories. The chlorine chemistry slightly increases the level of NO_2_ (by up to 0.038 ppbv) in the majority of the domain since (1) the production and the subsequent photolysis of ClNO_2_ recycles the NO_*x*_ and extends its lifetime, which increases both NO_2_ and NO (Figs. 7 and S5), and (2) the increased O_3_ level (Fig. 7) enhances the transformation of NO to NO_2_, which increases NO_2_ and decreases NO (Figs. 7 and S2). Some grid cells show a decrease in NO_2_ (as much as 0.09 ppbv) because the enhanced oxidative capacity (Sect. 3.3) promotes the cleansing of NO_2_ via OH, forming HNO_3_. The full halogen chemistry enhances NO_2_ (up to 0.20 ppbv) over the North Sea and the Mediterranean Sea and decreases NO_2_ (by as much as 1.7 ppbv) in the most polluted hot spots. The increase in NO_2_ occurs through the reactions of XO with NO. Meanwhile, in the most polluted regions, the NO–NO_2_ balance is predominantly controlled by the reactions of NO with HO_2_ and O_3_. With the decrease in HO_2_ and O_3_ due to the halogen chemistry, the transformation of NO to NO_2_ is reduced, which leads to decreasing NO_2_ and increasing NO.

The monthly average O_3_ concentration over Europe from the BASE simulation was relatively high (up to 57 ppbv), especially over southern Europe where higher temperatures and more intensive radiation promote the formation of this secondary pollutant. Chlorine chemistry increases O_3_ levels over the land of Europe with a maximum increment of 0.22 ppbv and decreases O_3_ over the oceanic area by as much as 0.76 ppbv. The full halogen chemistry decreases O_3_ throughout the domain, with a maximum reduction of 10 ppbv. On average, the halogen chemistry reduces the O_3_ concentration by more than 3.0 ppbv in coastal Europe and by over 2.0 ppbv over western and central Europe (nearly 1000 km from the ocean). Our model simulation highlights the fact that halogen chemistry has a large impact on O_3_ concentrations over oceanic areas and a moderate impact on O_3_ over coastal and continental regions of Europe.

Muñiz-Unamunzaga et al. (2018) reported a decrease of −2.0 ppbv O_3_ in the inland areas of the western US (several hundred kilometers from the ocean) and a reduction of 2.5 to 5.0 ppbv O_3_ in the coastal regions due to the full halogen chemistry. Sarwar et al. (2015) suggested that the inclusion of halogen processes reduced O_3_ concentrations by 2.0 to 4.0 ppbv over most of the terrestrial regions in the Northern Hemisphere and over 6.0 ppbv in some coastal areas. Sherwen et al. (2017) used a revised version of GEOS-Chem (Sherwen et al., 2016) with halogen chemistry to show substantial reductions in O_3_ over Europe, with an average reduction of 13.5 ppbv in the domain and a maximum of 28.9 ppbv in some locations.

### 3.5 Implications for policy assessment

The current air quality management in Europe has two main objectives: (1) to protect human health and (2) to protect the environment. While many plans and measures have prioritized particulate matter (PM) or NO_2_, policies to reduce O_3_ concentrations are still needed (EEA, 2018a). The World Health Organization (WHO) Air Quality Guidelines value for O_3_ (maximum daily 8 h mean of 100 µg m^−3^) was exceeded in 96% of all the reporting stations in Europe, although this is especially true for areas near the Mediterranean Sea. According to the European Environment Agency’s latest report, 12% of the EU-28 urban population is exposed to O_3_ concentrations above the European Union target value threshold (a maximum daily 8 h mean of 120 µg m^−3^ is not to be exceeded on more than 25 d yr^−1^, as set out by the Directive 2008/50/EC) in 2016. Apart from significant potential health effects (Jerrett et al., 2009; Malley et al., 2017), O_3_ is also known to have a negative impact on vegetation (Mills et al., 2011). The target value for the protection of vegetation (18 000 µg m^−3^ h^−1^ accumulated over May to July), based on the AOT40 index (Accumulated Ozone exposure over a Threshold of 40 ppbv), was exceeded for about 31% of all agricultural land in all European countries. The critical level for this pollutant (10 000 µg m^−3^ h^−1^ accumulated over April to September) was exceeded in 60% of the total forest area of the continent in 2016 (EEA, 2018a).

**Figure 8.**
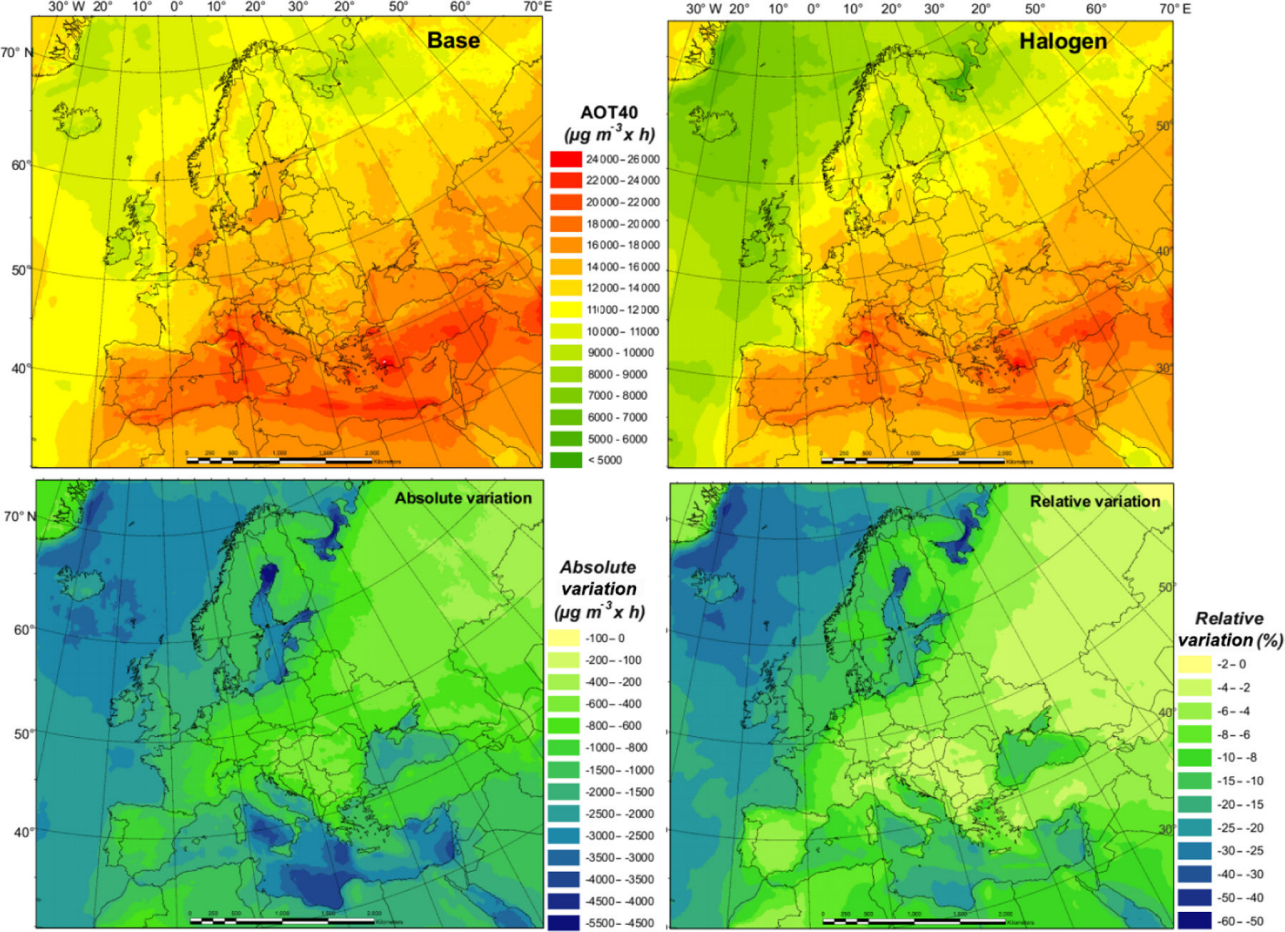
AOT40 for July in the BASE and HAL simulations and absolute and relative changes between the two simulations.

Since our experiment covers only 1 month, it is not possible to assess the impact of halogen chemistry on these two indexes. Nonetheless, we have compared the results of both maximum daily 8 h mean and 1-month AOT40 (over July; Fig. 8) for the BASE and HAL simulations. The relative variation provides a good indication of the potential impact of considering halogens in our modeling system in the estimation of legally relevant indexes.

We find that halogen chemistry strongly affects the ambient O_3_ concentration and may need to be considered in the formulation of plans and strategies for O_3_ non-attainment areas. We see differences between BASE and HAL simulations (over land in July 2016) as high as 12% and 36% for the number of days with daily maximum 8 h O_3_ over 120 µg m^−3^ and the monthly average daily maximum 8 h O_3_ level, respectively (Figs. S6 and S7). Furthermore, we notice strong regional differences, mainly between coastal and inland areas. The considerable effect of halogen chemistry on air quality implies the need to improve the robustness and accuracy of modeling tools to design customized policies to control O_3_.

In Sect. 3.3 and 3.4, we have also discussed the effect of halogen chemistry on the partitioning of OH/HO_2_ and NO/NO_2_. The budgets of HO_*x*_ and NO_*x*_ are key parameters to accurately simulate the formation of O_3_ and its response to the reduction of precursors, namely NO_*x*_ and VOCs (e.g., Li et al., 2018). Air quality models are predominantly used to formulate air pollution control policy by examining the responses of O_3_ levels to various reduction rates of NO_*x*_ and/or VOCs. The models that do not include the comprehensive halogen chemistry can potentially lead to different O_3_ concentration responses to NO_*x*_ and/or VOC emission changes in Europe.

This study also demonstrates that chlorine chemistry affects the formation of O_3_. The current policy is only designed to control the long-lived chlorinated species (Hossaini et al., 2015) but not reactive chlorine species, e.g., HCl, chloride, and short-lived chlorocarbons, from coal burning, biomass burning, and industrial activities. The coal-fired power plants in the EU (EEA, 2018b; Kuklinska et al., 2015) can potentially provide chlorine sources, making the implications of halogen chemistry even more relevant.

## 4 Conclusion

We applied the CMAQ model with comprehensive halogen chemistry (Cl, Br, and I) to conduct high-resolution simulations for examining the impact of halogen chemistry on air quality over Europe.

The comparison of model results with observations from over 400 monitoring sites indicates that the CMAQ model is capable of reproducing the concentrations and temporal variations of air pollutants over Europe and can be employed to study the impact of halogen chemistry in Europe. The comparison of predicted halogen species concentrations with measurements suggests that the CMAQ model is able to predict observed levels of chlorine and iodine species, although it underestimates bromine species.

Chlorine chemistry enhances the atmospheric oxidation capacity by significantly increasing the level of the Cl radical and affects the levels of OH, HO_2_, NO_3_, O_3_, and NO_2_. The combined halogen chemistry marginally increases the level of OH and reduces HO_2_, NO_3_, and O_3_. The impact of halogen chemistry on the ambient concentration of NO_2_ is smaller but non-negligible.

Halogen chemistry significantly influences the atmospheric oxidation capacity throughout the day by imposing the highest effect on Cl in the early morning, maximum effects on OH and HO_2_ in daytime, and the largest effect on NO_3_ at night. Halogen chemistry can have a strong influence on atmospheric composition over oceanic and coastal regions but also some noticeable impacts over continental Europe. This study highlights the potential benefit of incorporating halogen chemistry into air quality models for policy development.

Although the incorporation of the halogen chemistry may improve the capabilities of 3-D Eulerian chemical transport models, we acknowledge that large uncertainties still exist in the assessment of the halogen chemistry impact due to emission inventories (e.g., chlorine emission inventory; Wang et al., 2019), model configurations (e.g., grid size; Sommariva et al., 2018), and chemical mechanisms (e.g., photolysis rate of iodine oxides, recycling rate of halogen species on aerosol; Simpson et al., 2015). Further field, laboratory, and theoretical studies are needed to constrain modeling studies for evaluating the impacts of halogen chemistry on air quality and for assessing air quality policy implications.

## Supplementary Material

Supplement1

## Figures and Tables

**Table 1.** Statistical summary of model performance.

Statistics	O_3_ (µg m^−3^)				NO_2_ (µg m^−3^)			
	Coastal		Inland		Coastal		Inland	
	Base	HAL	Base	HAL	Base	HAL	Base	HAL
MB	−0.9	−6.8	−2.3	−6.6	−3.7	−3.7	−3.1	−3.1
ME	16.9	17.5	19.7	20.2	5.8	5.8	5.7	5.6
RMSE	22.6	23.1	25.8	26.4	7.7	7.7	7.2	7.2
*r*	0.65	0.67	0.60	0.61	0.44	0.44	0.42	0.42
IOA	0.64	0.62	0.62	0.61	0.31	0.31	0.18	0.19

MB: mean bias, RMSE: root mean square error, *r*: correlation coefficient, IOA: index of agreement.

**Table 2.** Simulated average concentrations of inorganic halogen species over the ocean.

Species	Concentration (ppt)	Percentage (%)	Species	Concentration (ppt)	Percentage (%)	Species	Concentration (ppt)	Percentage (%)
HCl	247.9	96.1	HOBr	1.06	27.0	HOI	5.1	52.0
ClNO_2_	4.9	1.9	BrCl	0.71	18.2	INO_3_	2.8	28.7
HOCl	3.8	1.5	BrNO_3_	0.67	16.9	IO	1.0	10.3
ClNO_3_	1.2	0.5	HBr	0.66	16.8	I	0.4	4.0
ClO	0.25	0.1	BrO	0.38	9.7	I_2_O_3_	0.3	3.1
Cl_2_	0.02	0.008	Br_2_	0.33	8.4	HI	0.1	1.0
Cl	0.0002	0.0001	BrNO_2_	0.09	2.4	I_2_	0.05	0.5
–			Br	0.03	0.7	INO	0.03	0.3
–			–			I_2_O_2_	0.01	0.1
–			–			INO_2_	0.01	0.1
–			–			I_2_O_4_	0.004	< 0.1

Total Cl	258.1	100	Total Br	3.9	100	Total I	9.8	100

**Table 3.** Comparison of observed and simulated halogen species.

Location	Species	Observation^a^	Simulation^b^
Hessen, Germany^c^	ClNO_2_	800.0	273.4
London, United Kingdom^d^	ClNO_2_	724.0	801.5
Weybourne, United Kingdom^e^	ClNO_2_	65	373
Weybourne, United Kingdom^f^	ClNO_2_	946	373
Weybourne, United Kingdom^g^	ClNO_2_	1100 (summer), 75.6 (autumn), 733 (winter)	373
Leicester, United Kingdom^g^	ClNO_2_	274 (spring), 74.2 (summer), 248 (winter)	274
Penlee Point, United Kingdom^g^	ClNO_2_	922	319
Mace Head, Ireland^h^	BrO	6.5	10.1
Brittany, France^i^	BrO	7.5	0.4
Dead Sea^j^	BrO	100.0	0.2
Mace Head, Ireland^k^	IO	4.0–50.0	3.9
Brittany, France^l^	IO	7.7–30.0	1.1
Dagebüll, Germany^m^	IO	2.0	9.0
Atlantic Ocean^n^	IO	0.4 to 0.5 (daytime average)	0.4 to 2.0 (daytime average)

a Maximum value (pptv).

b Maximum value (pptv) from the HAL simulation.

c Phillips et al. (2012).

d Bannan et al. (2015).

e Bannan et al. (2017).

f Sherwen et al. (2017).

g Sommariva et al. (2018).

h Saiz-Lopez et al. (2004).

i Mahajan et al. (2009).

j Matveev et al. (2001), Holla et al. (2015).

k Allan et al. (2000), Commane et al. (2011).

l Britter et al. (2005), Furneaux et al. (2010).

m Peters et al. (2005).

n Prados-Roman et al. (2015)
