# Supplementary material for: Impact of halogen chemistry on summertime air quality in coastal and continental Europe: application of the CMAQ model and implications for regulation
Source: Atmos Chem Phys. Author manuscript; Available in PMC 2020 May 18. (PMC7232855; doi:10.5194/acp-19-15321-2019)
Supplement: Supplement1 [file NIHMS1559395-supplement-Supplement1.pdf]

Supplement of

Impact of halogen chemistry on summertime air quality in coastal and continental Europe: application of CMAQ model and implication for regulation

Qinyi Li[1], Rafael Borge[2], Golam Sarwar[3], David de la Paz[2], Brett Gantt[4], Jessica Domingo[2], Carlos A. Cuevas[1], and Alfonso Saiz-Lopez[1]*

1 Department of Atmospheric Chemistry and Climate, Institute of Physical Chemistry Rocasolano, CSIC, Madrid 28006, Spain
2 Environmental Modelling Laboratory, Department of Chemical & Environmental Engineering, Universidad Politécnica de Madrid (UPM), Madrid, Spain
3 National Exposure Research Laboratory, Environmental Protection Agency, Research Triangle Park, NC 27711, United States
4 Office of Air Quality Planning and Standards, Environmental Protection Agency, Research Triangle Park, NC 27711, United States

*Correspondence to: Alfonso Saiz-Lopez (a.saiz@csic.es)

Table S1: Chlorine chemistry used in the study

Reaction level	Reaction	Rate Expression	Ref
Cl01	Cl + O3 = ClO + O2	$k=2.3 \times 10^{-11} e^{(-200/T)}$	1
Cl02	ClO + ClO = 0.3 Cl2 + 1.4 Cl + O2	$k=1.63 \times 10^{-14}$	1
Cl03	ClO + NO = Cl + NO2	$k=6.4 \times 10^{-12} e^{(290/T)}$	1
Cl04	ClO + HO2 = HOCl + O2	$k=2.7 \times 10^{-12} e^{(220/T)}$	1
Cl05	ClO + NO2 = ClNO3	$k=\{k_o[M]/(1+k_o[M]/k_\infty)\} F^z$<br>$k_o= 1.8 \times 10^{-31}(T/300)^{-3.4}$<br>$k_\infty= 1.5 \times 10^{-11}(T/300)^{-1.9}$<br>F = 0.6 and N = 1.0<br>$Z=\{(1/N)+\log_{10}[k_o [M]/k_\infty]^2\}^{-1}$	2
Cl06	Cl + NO2 = ClNO2	$k=\{k_o[M]/(1+k_o[M]/k_\infty)\} F^z$<br>$k_o= 1.8 \times 10^{-31}(T/300)^{-2.0}$<br>$k_\infty= 1.0 \times 10^{-10}(T/300)^{-1.0}$<br>F = 0.6 and N = 1.0<br>$Z=\{(1/N)+\log_{10}[k_o [M]/k_\infty]^2\}^{-1}$	1
Cl07	HCl + OH = Cl + H2O	$k=6.58 \times 10^{-13}(T/300)^{1.16} e^{(-58/T)}$	1
Cl08	FMCl + OH = Cl + CO + H2O	$k=3.67 \times 10^{-11} e^{(-1419/T)}$	1
Cl09	ClO + MEO2 = Cl + FORM + HO2	$k=4.1 \times 10^{-13} e^{(-800/T)}$	1
Cl10	CH4 + Cl = HCl + MEO2	$k=6.6 \times 10^{-12} e^{(-1240/T)}$	1
Cl11	PAR + Cl = HCl	$k=5.00 \times 10^{-11}$	1,3
Cl12	ETHA + Cl = HCl + 0.991 ALD2 + 0.991 XO2 + 0.009 XO2N + HO2	$k=8.3 \times 10^{-11} e^{(-100/T)}$	1
Cl13	ETH + Cl = FMCl + 2.0 XO2 + HO2 + FORM	$k=1.07 \times 10^{-10}$	1
Cl14	OLE + Cl = FMCl + 0.33 ALD2 + 0.67 ALDX + 2.0 XO2 + HO2 – PAR	$k=2.5 \times 10^{-10}$	1
Cl15	IOLE + Cl = 0.3 HCl + 0.7 FMCl + 0.45 ALD2 + 0.55 ALDX + 0.3 OLE + 0.3 PAR + 1.7 XO2 + HO_2	$k=3.5 \times 10^{-10}$	1
Cl16	ISOP + Cl = 0.15 HCl + XO2 + HO2 + 0.85	$k=4.3 \times 10^{-10}$	1

	FMCl + ISPD		
Cl17	FORM + Cl = HCl + HO2 + CO	k=8.2 × 10^{-11} $e^{(-34/T)}$	1
Cl18	ALD2 + Cl = HCl + C2O3	k=7.9 × 10^{-11}	1
Cl19	ALDX + Cl = HCl + CXO3	k=1.3 × 10^{-10}	1
Cl20	MEOH + Cl = HCl + HO2 + FORM	k=5.5 × 10^{-11}	1
Cl21	ETOH + Cl = HCl + HO2 + ALD2	k=8.2 × 10^{-11} $e^{(45/T)}$	1
Cl22	TOL + Cl = HCl + 0.88 XO2 + 0.88 HO2 + 0.12 XO2N	k=6.1 × 10^{-11}	1
Cl23	XYL + Cl = HCl + 0.84 XO2 + 0.84 HO2 + 0.16 XO2N	k=1.2 × 10^{-10}	1
Cl24	NAPH + Cl = HCl + 0.84 XO2 + 0.84 HO2 + 0.16 XO2N	k=1.2 × 10^{-10}	1
Cl25	Cl2 = 2 Cl	Photolysis	1
Cl26	HOCl = OH + Cl	Photolysis	1
Cl27	FMCl = Cl + CO + HO2	Photolysis	1
Cl28	ClNO2 = Cl + NO2	Photolysis	1
Cl29	ClNO3 = ClO + NO2	Photolysis	4
Cl30	ClNO3 = Cl + NO3	Photolysis	4
HET_N2O5	N_2O_5 (+ H_2O) → Y × (HNO3 + $ClNO_2$) + 2 × (1 – Y) HNO_3	$k=\left(\frac{\tilde{d}}{2D}+\frac{4}{\acute{c}\gamma}\right)^{-1}A$ γ = f(aerosol composition)	5
HET_ClNO3_WA	$ClNO_3$ (+ H_2O)→ HOCl + HNO_3	$k=\left(\frac{\tilde{d}}{2D}+\frac{4}{\acute{c}\gamma}\right)^{-1}A$ γ= 0.024	6

Note:

Cl2 = molecular chlorine, Cl = atomic chlorine, HOCl = hypochlorous acid, ClNO2 = nitryl chloride, ClNO3 = chlorine nitrate, HCl = hydrochloric acid, OH = hydroxyl radical, O2 = oxygen, O3 = ozone, ClO = chlorine oxide, NO = nitric oxide, NO2 = nitrogen dioxide, H2O = water vapor, HO2 = hydroperoxy radical, FMCl = formyl chloride, CO = carbon monoxide, CH4 = methane, ETHA = ethane, MEO2 = methylperoxy radical, PAR = paraffin carbon bond, XO2 = NO-to-NO_2 operator, XO2N = NO-to-nitrate operator, FORM = formaldehyde, ALD2 = acetaldehyde, ALDX = propionaldehyde and higher aldehydes, OLE = terminal olefinic carbon bond, IOLE = internal olefinic carbon bond, ETH = ethene, ISOP = isoprene, ISPD = isoprene product, MEOH = methanol, ETOH = ethanol, C2O3 = acetylperoxy radical, CXO3 = higher acylperoxy radicals, TOL = toluene, XYL = xylene, NAPH = napthalene, T= temperature, k = rate constant, M = total pressure, $\tilde{d}$= effective diameter, D = diffusivity in air, $\acute{c}$= mean molecular velocity, A = aerosol surface area concentration, γ= reactive uptake coefficient, Y = yield of ClNO2 (7). Cl01-Cl30 are gas-phase reactions while HET_N2O5 and HET_ClNO3_WA are heterogeneous reactions.

Ref: 1 - Sarwar, et al. 2019; 2 - Sander, et al., 2011; 3- Yarwood et al., 2014; 4 – Atkinson et el., 2007; 5 – Davis et al., 2008; 6 – Deiber et al., 2004; 7 – Bertram et al., 2009.

Table S2. Setup for the model simulations.

Category	Setup WRF simulation
Shortwave radiation	MM5 Shortwave radiation scheme (Dudhia, 1988)
Longwave radiation	Eta Geophysical Fluid Dinamics Laboratory (GFDL) (Schwarzkopf and Fels, 1991)
Land-surface model	Noah LSM (Chen and Dudhia, 2001)
Microphysics scheme	WSM6 (Hong and Lim, 2006)
PBL scheme	BULK: Yonsei University (YSU) (Hong et al., 2006)
Horizontal resolution	12 km
Vertical resolution	35 sigma levels (Lowest level ≈ 20 m).<br>Sigma (σ) levels: 1.000, 0.998, 0.995, 0.990, 0.985, 0.980, 0.970, 0.960, 0.950, 0.940, 0.930, 0.920, 0.910, 0.900, 0.880, 0.860, 0.840, 0.820, 0.800, 0.770, 0.740, 0.700, 0.650, 0.600, 0.550, 0.500, 0.450, 0.400, 0.350, 0.300, 0.250, 0.200, 0.150, 0.100, 0.050, 0.000.

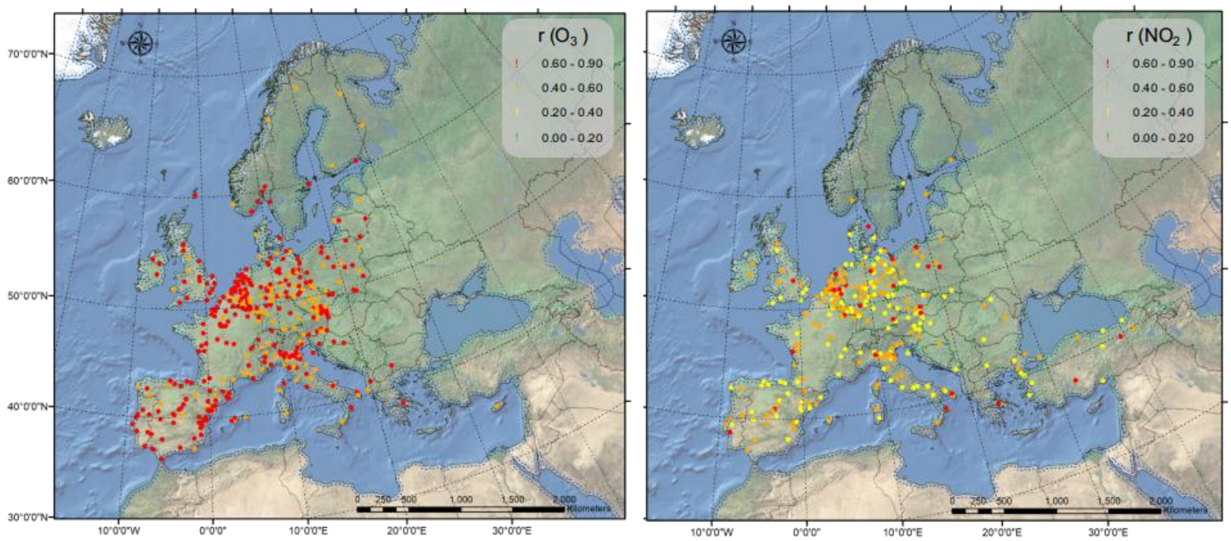

Figure S1. Correlation coefficient of observed and predicted (HAL simulation) hourly O_3 and NO_2

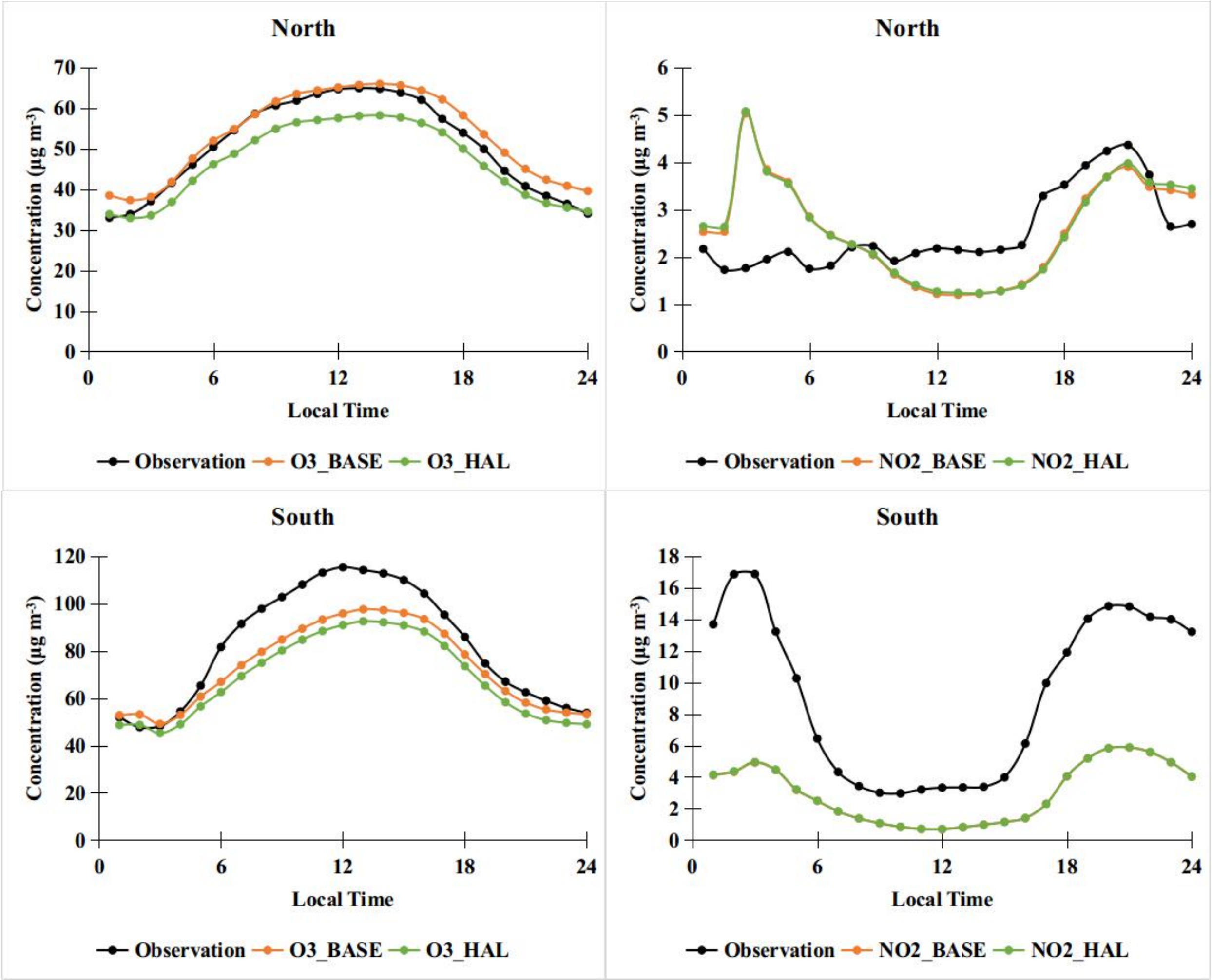

Figure S2. Diurnal variation of observed and simulated (BASE and HAL) O_3 and NO_2 over northern (>50°N) and southern (<50°N) Europe.

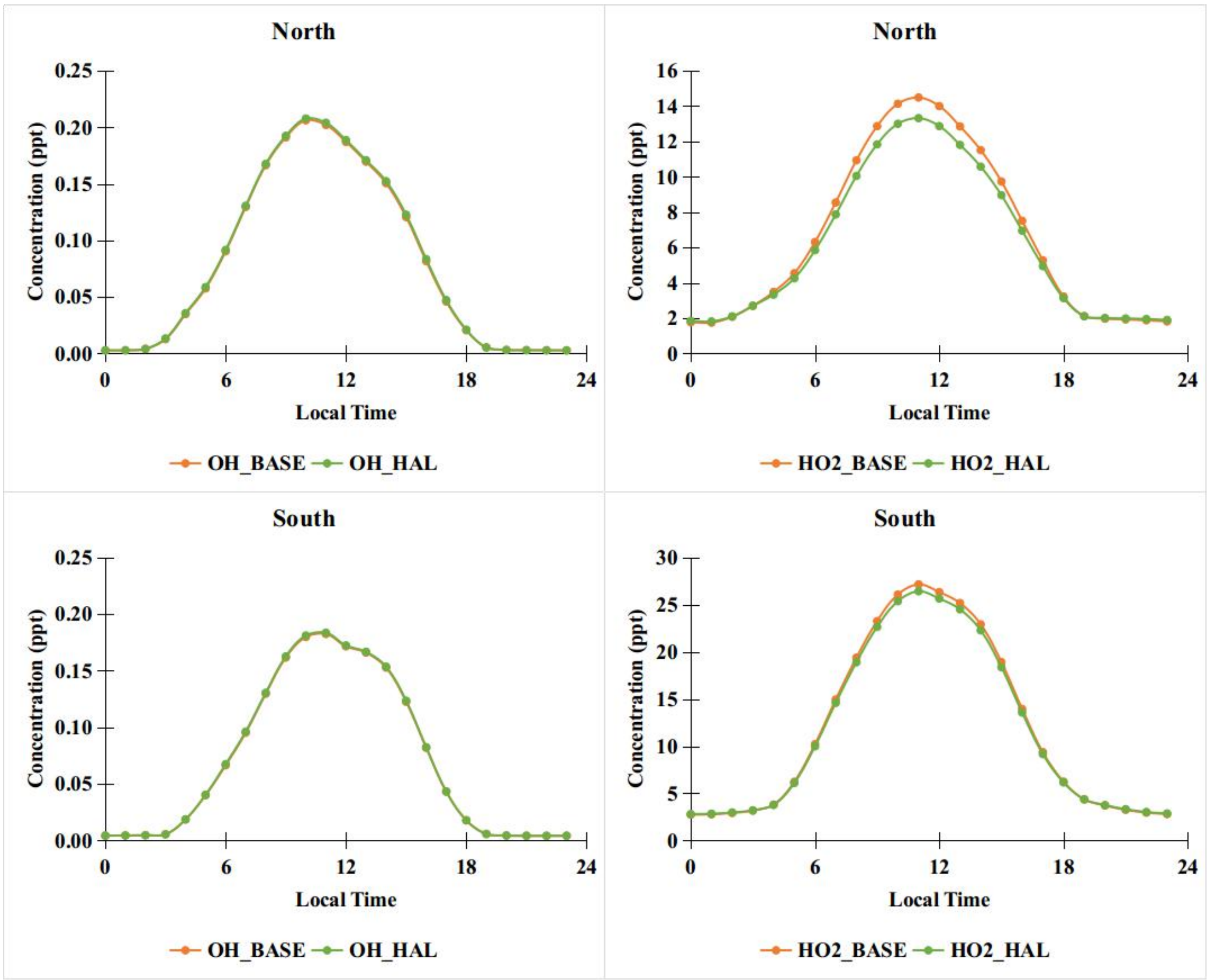

Figure S3. Diurnal variation of simulated (BASE and HAL) OH and HO_2 over northern and southern Europe.

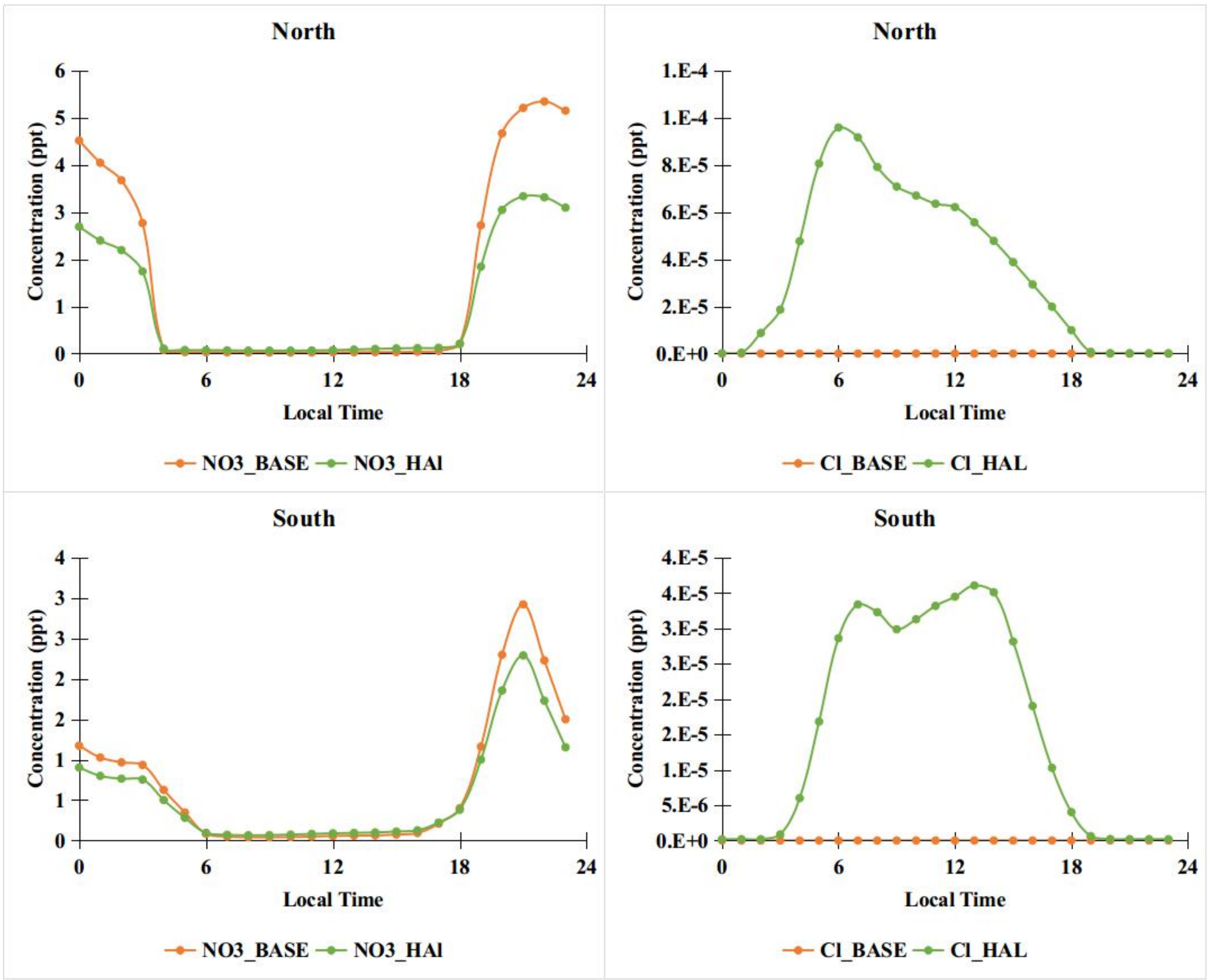

Figure S4. Diurnal variation of simulated (BASE and HAL) NO_3 and Cl over northern and southern Europe.

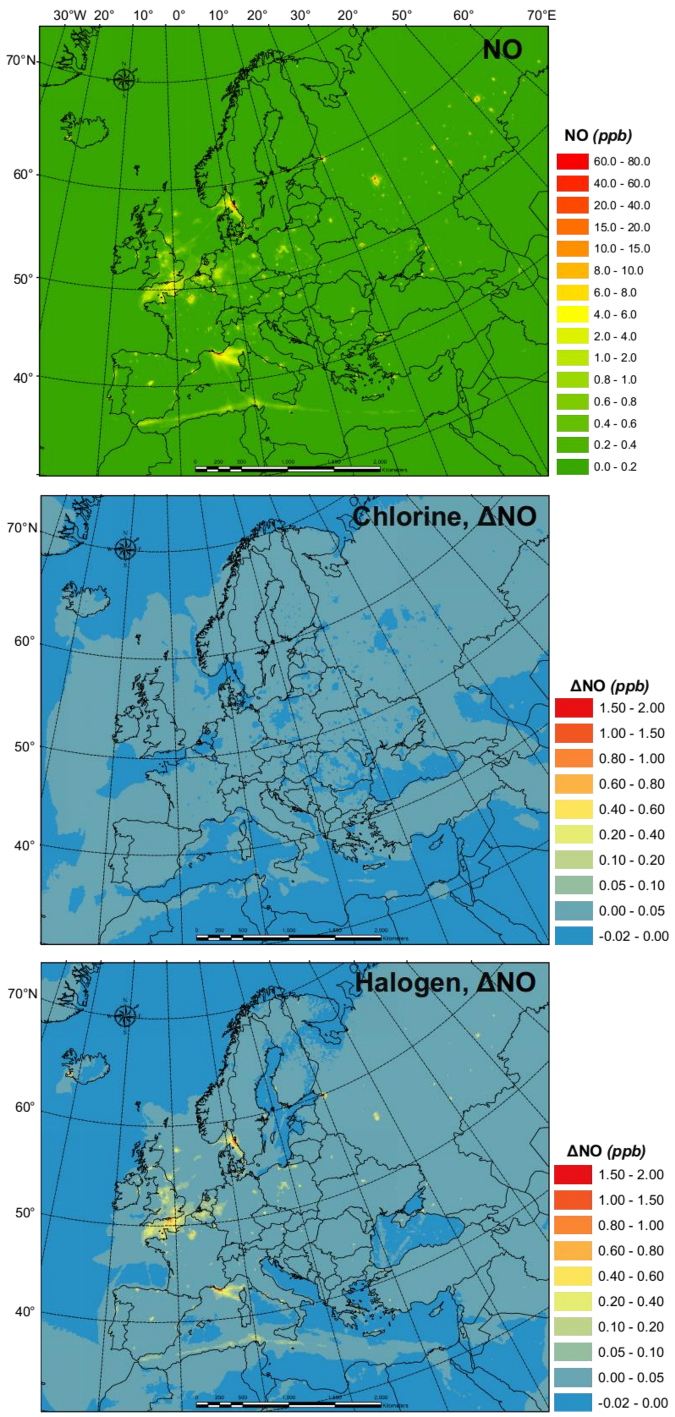

Figure S5. Monthly average NO concentration in the BASE simulation, and changes induced by chlorine (CL) and full halogen chemistry (HAL).

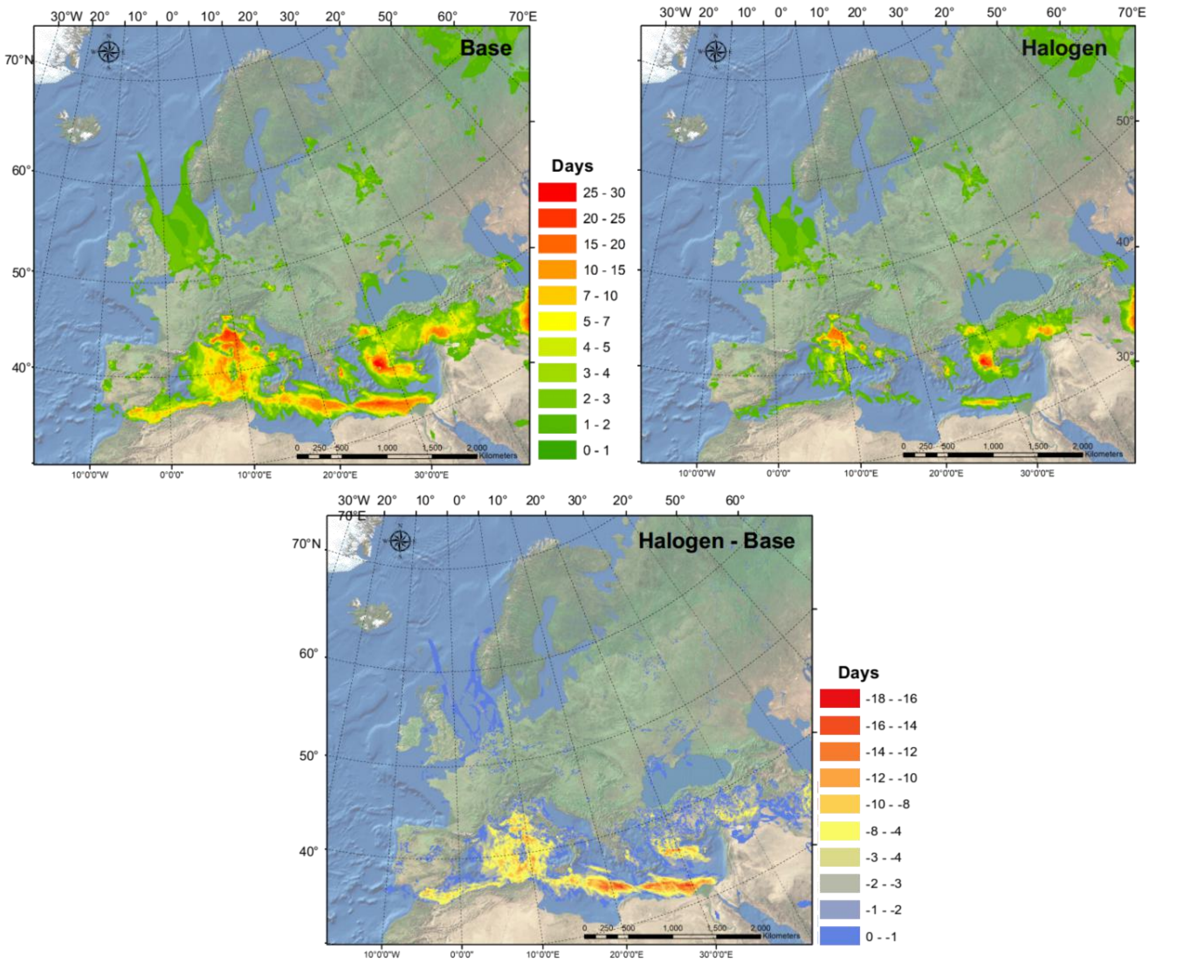

Figure S6. Number of days with daily maximum 8 h O_3 concentration over 120 µg·m^{-3} in BASE and HAL simulations, and the absolute variation between the two simulations.

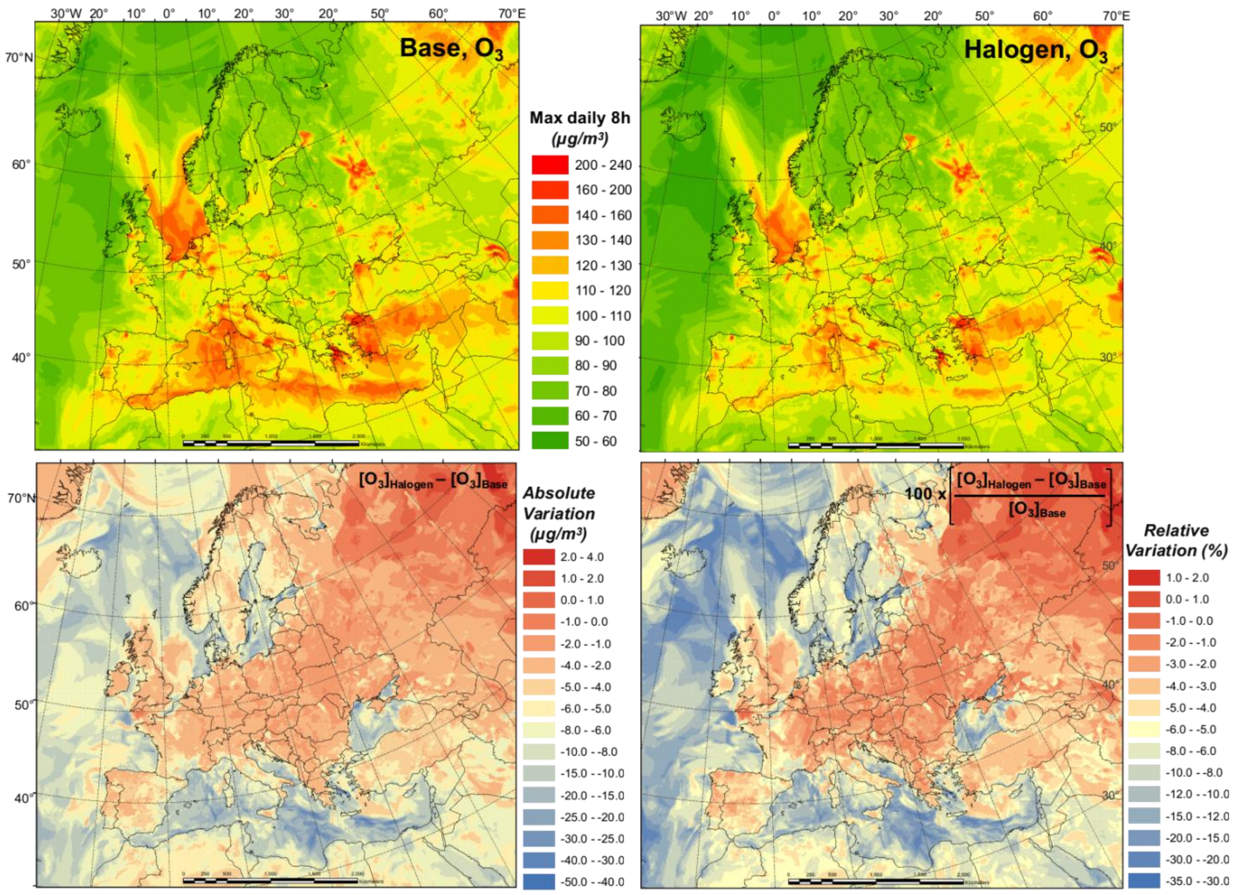

Figure S7. Monthly average of daily maximum 8 h O_3 concentrations in BASE and HAL simulations, and the absolute and relative changes between the two simulations.

Reference:

Atkinson, R.; Baulch, D. L.; Cox, R. A.; Crowley, J. N.; Hampson, R. F.; Hynes, R. G.; Jenkin, M. E.; Rossi, M. J.; and Troe, J.: Evaluated kinetic and photochemical data for atmospheric chemistry: Volume III – gas phase reactions of inorganic halogens. Atmos. Chem. Phys., 7, 981-1191, 2007.

Bertram, T. H.; Thornton, J.A.: Toward a general parameterization of N2O5 reactivity on aqueous particles: the competing effects of particle liquid water, nitrate and chloride. Atmos. Chem. Phys, 2009, 9, 8351–8363, doi:10.5194.

Chen, F. and Dudhia, J.: Coupling an Advanced Land Surface–Hydrology Model with the Penn State–NCAR MM5 Modeling System. Part II: Preliminary Model Validation, Mon. Weather Rev., 129(4), 587–604, doi:10.1175/1520-0493(2001)129<0587:CAALSH>2.0.CO;2, 2001.

Davis, J. M.; Bhave, P. V.; Foley, K. M.: Parameterization of N2O5 reaction probabilities on the surface of particles containing ammonium, sulfate, and nitrate. Atmos. Chem. Phys., 8, 5295–5311, 2008.

Deiber, G.; George, C.; Calvé, S.; Schweitzer, F.; Mirabel, P.: Uptake study of ClONO2 and BrONO2 by Halide containing droplets. Atmos. Chem. Phys., 4, 1291–1299, doi:10.5194/acp-4-1291-2004, 2004.

Dudhia, J.: Numerical Study of Convection Observed during the Winter Monsoon Experiment Using a Mesoscale Two-Dimensional Model, J. Atmos. Sci., 46(20), 3077–3107, doi:10.1175/1520-0469(1989)046<3077:NSOCOD>2.0.CO;2, 1988.

Hong, S.-Y. and Lim, J.-O. J.: The WRF Single-Moment 6-Class Microphysics Scheme (WSM6), J. Korean Meteorol. Soc., 42(2), 129–151, 2006.

Hong, S.-Y., Noh, Y. and Dudhia, J.: A New Vertical Diffusion Package with an Explicit Treatment of Entrainment Processes, Mon. Weather Rev., 134(9), 2318–2341, doi:10.1175/MWR3199.1, 2006.

Sander, S. P.; Friedl, R. R.; Abbatt, J. P. D.; Barker, J. R.; Burkholder, J. B.; Golden, D. M.; Kolb, C. E.; Kurylo, M. J.; Moortgat, G. K.; Wine, P. H.; Huie, R. E.; Orkin, V. L.: Chemical kinetics and photochemical data for use in atmospheric studies, Evaluation Number 17, Tech. rep., NASA Jet Propulsion Laboratory, Pasadena, 2011.

Sarwar, G.; Simon, H.; Bhave, P; G. Yarwood, G.: Examining the impact of heterogeneous nitryl chloride production on air quality across the United States. Atmos. Chem. Phys, 2012, 12, 1-19.

Schwarzkopf, M. D. and Fels, S. B.: The simplified exchange method revisited: An accurate, rapid method for computation of infrared cooling rates and fluxes, J. Geophys. Res. Atmos., 96(D5), 9075–9096, doi:10.1029/89JD01598, 1991.

Yarwood, G.; , Sakulyanontvittaya, T.; Nopmongcol, O.; Koo, B.: Ozone depletion by bromine and iodine over the Gulf of Mexico, Final Report to the Texas Natural Resource Conservation Commission, November, 2014.
